# Supplementary material for: Classification of Vascular Plants in Vietnam According to Modern Classification Systems
Source: Plants (Basel). 2023 Feb 20;12(4):967. doi: 10.3390/plants12040967 (PMC9966254; doi:10.3390/plants12040967)
Supplement: Supplementary file 1 [file plants-12-00967-s001.zip › plants-2038571-supplementary.pdf]

**Table S1.** Comparison of classifications of lycophyte and fern families of Vietnam in IFV and CPSV. (Scientific names in boldface were newly circumscribed for Vietnam).

| **IFV (1999)** | | **CPSV (2001)** | | **Phan (2010)** | |
|---|---|---|---|---|---|
| Families | Genera | Families | Genera | Families | Genera |
| | | **Psilotophyta** | | **Psilotopsida** | |
| | | | | 1. Ophioglossaceae | *Botrychium* |
| | | | | | *Ophioglossum* |
| 1. Psilotaceae | *Psilotum* | 1. Psilotaceae | *Psilotum* | 2. Psilotaceae | *Psilotum* |
| | | **Lycopodiophyta** | | | |
| 2. Lycopodiaceae | *Huperzia* | 1. Lycopodiaceae | *Huperzia* | | |
| | | | *Lycopodiella* | | |
| | *Lycopodium* | | *Lycopodium* | | |
| 3. Isoetaceae | *Isoetes* | 2. Isoetaceae | *Isoetes* | | |
| 4. Selaginellaceae | *Selaginella* | 3. Selaginellaceae | *Selaginella* | | |
| | | **Equisetophyta** | | **Equisetopsida** | |
| 5. Equisetaceae | *Equisetum* | 1. Equisetaceae | *Equisetum* | 3. Equisetaceae | *Equisetum* |
| | | | | **Marattiopsida** | |
| | | | | 4. Marattiaceae | *Angiopteris* |
| | | | | | ***Christensenia*** |
| | | | | | *Marattia* |
| | | **Polypodiophyta** | | **Polypodiopsida** | |
| 6. Adiantaceae | *Adiantum* | 1. Adiantaceae | *Adiantum* | | |
| | *Cheilanthes* | | *Cheilanthes* | | |
| | *Coniogramme* | | *Coniogramme* | | |
| | *Dryopteris* | | *Dryopteris* | | |
| | *Hemionitis* | | *Hemionitis* | | |
| | | | *Mildella* | | |
| | | | *Monachosorum* | | |
| | *Onychium* | | *Onychium* | | |
| | *Pellaea* | | *Pellaea* | | |
| | *Pityrogramma* | | *Pityrogramma* | | |
| | *Syngramma* | | *Syngramma* | | |
| | *Taenitis* | | *Taenitis* | | |
| | *Notholaena* | | | | |
| 7. Aspleniaceae | *Asplenium* | 2. Aspleniaceae | *Asplenium* | 19. Aspleniaceae | *Asplenium* |
| | | | | | ***Hymenoasple-nium*** |
| | *Athyrium* | | | | |
| | *Cystopteris* | | | | |
| | *Diplazium* | | | | |
| 8. Azollaceae | *Azolla* | 3. Azollaceae | *Azolla* | | |
| 9. Blechnaceae | *Blechnum* | 4. Blechnaceae | *Blechnum* | 22. Blechnaceae | *Blechnum* |
| | *Brainea* | | *Brainea* | | *Brainea* |
| | | | *Stenochlaena* | | *Stenochlaena* |
| | *Woodwardia* | | *Woodwardia* | | *Woodwardia* |
| 10. Cheiropleu-riaceae | *Cheiropleuria* | 5. Cheiropleuriaceae | *Cheiropleuria* | | |
| 11. Cyathaceae | *Cyathea* | 6. Cyathaceae | *Cyathea* | 15. Cyatheaceae | *Cyathea* |
| | | | | | *Alsophila* |
| | | | | | *Sphaeropteris* |

| **IFV (1999)** | | **CPSV (2001)** | | **Phan (2010)** | |
|---|---|---|---|---|---|
| Families | Genera | Families | Genera | Families | Genera |
| 12. Davalliaceae | *Araiostegia* | 7. Davalliaceae | *Araiostegia* | 27. Davalliaceae | *Araiostegia* |
| | *Arthropteris* | | | | |
| | *Davallia* | | *Davallia* | | *Davallia* |
| | *Davallodes* | | *Davallodes* | | *Davallodes* |
| | *Humata* | | | | |
| | *Gymnogrammitis* | | *Gymnogrammitis* | | |
| | *Nephrolepis* | | | | |
| | *Leucostegia* | | *Leucostegia* | | |
| | *Rhumohra* | | *Rhumohra* | | |
| | *Oleandra* | | | | |
| 13. Dennstaed-tiaceae | *Dennstaedtia* | 8. Dennstaedtiaceae | *Dennstaedtia* | 17. Dennstaedtiaceae | *Dennstaedtia* |
| | *Histiopteris* | | *Histiopteris* | | *Histiopteris* |
| | *Hypolepis* | | *Hypolepis* | | *Hypolepis* |
| | *Microlepia* | | *Microlepia* | | *Microlepia* |
| | *Monachosorium* | | | | *Monachosorium* |
| | *Pteridium* | | *Pteridium* | | *Pteridium* |
| | *Lindsaea* | | *Lindsaea* | 16. **Lindsaeaceae** | *Lindsaea* |
| | *Sphenomeris* | | *Sphenomeris* | | *Sphenomeris* |
| | *Tapeinidium* | | *Tapeinidium* | | *Tapeinidium* |
| 14. Thyrsopterida-ceae | *Cibotium* | 9. Dicksoniaceae | *Cibotium* | 14. **Cibotiaceae** | *Cibotium* |
| 15. Dipteridaceae | | 10. Dipteridaceae | | 8. Dipteridaceae | *Cheiropleuria* |
| | *Dipteris* | | *Dipteris* | | *Dipteris* |
| 16. Dryopterida-ceae | | 11. Dryopteridaceae | *Acrophorus* | 23. Dryopteridaceae | *Acrophorus* |
| | | | | | ***Acrorumohra*** |
| | *Arachniodes* | | *Arachniodes* | | *Arachniodes* |
| | *Ctenitis* | | *Ctenitis* | | *Ctenitis* |
| | *Ctenitopsis* | | | | |
| | *Cyrtomium* | | *Cyrtomium* | | *Cyrtomium* |
| | *Cyrtogonellum* | | | | *Cyrtogonellum* |
| | *Diacalpe* | | *Diacalpe* | | |
| | *Didymochlaena* | | *Didymochlaena* | | *Didymochlaena* |
| | *Dryopteris* | | *Dryopteris* | | *Dryopteris* |
| | | | | | *Elaphoglossum* |
| | | | | | *Hypodematium* |
| | | | | | *Leucostegia* |
| | | | | | *Lomagramma* |
| | | | | | *Peranema* |
| | *Hemigramma* | | | | |
| | *Quercifilix* | | | | |
| | | | *Polystichopsis* | | |
| | *Polystichum* | | *Polystichum* | | *Polystichum* |
| | *Cyclopeltis* | | *Cyclopeltis* | 24. Lomariopsidaceae | *Cyclopeltis* |
| | | | *Heterogonium* | 25. Tectariaceae | *Heterogonium* |
| | | | *Pleocnemia* | | *Pleocnemia* |
| | *Pteridrys* | | *Pteridrys* | | *Pteridrys* |
| | *Tectaria* | | *Tectaria* | | *Tectaria* |
| 17. Gleicheniaceae | *Dicranopteris* | 12. Gleicheniaceae | *Dicranopteris* | 7. Gleicheniaceae | *Dicranopteris* |

| **IFV (1999)** | | **CPSV (2001)** | | **Phan (2010)** | |
|---|---|---|---|---|---|
| Families | Genera | Families | Genera | Families | Genera |
| | *Diplopterygium* | | *Diplopterygium* | | *Diplopterygium* |
| | *Gleichenia* | | *Gleichenia* | | *Gleichenia* |
| 18. Grammitidaceae | *Acrosorus* | 13. Grammitidaceae | *Acrosorus* | | |
| | *Calymmodon* | | *Calymmodon* | | |
| | *Ctenopteris* | | *Ctenopteris* | | |
| | *Grammitis* | | *Grammitis* | | |
| | *Prosaptia* | | *Prosaptia* | | |
| | *Scleroglossum* | | *Scleroglossum* | | |
| | *Xiphopteris* | | *Xiphopteris* | | |
| | *Loxogramme* | | | | |
| 19. Hymenophyllaceae | *Cephalomanes* | 14. Hymenophyllaceae | *Cephalomanes* | 6. Hymenophyllaceae | *Cephalomanes* |
| | | | | | ***Abrodictyum*** |
| | *Crepidomanes* | | *Crepidomanes* | | *Crepidomanes* |
| | | | | | *Didymoglossum* |
| | *Gnocormus* | | | | |
| | *Hymenophyllum* | | *Hymenophyllum* | | *Hymenophyllum* |
| | *Mecodium* | | | | |
| | *Meringium* | | | | |
| | *Microgonium* | | | | |
| | *Sphaerocionium* | | *Sphaerocionium* | | |
| | *Trichomanes* | | *Trichomanes* | | *Trichomanes* |
| | *Vandenboschia* | | | | *Vandenboschia* |
| 20. Lomariopsidaceae | *Bolbitis* | 15. Lomariopsidaceae | *Bolbitis* | | |
| | *Egenolfia* | | | | |
| | *Elaphoglossum* | | *Elaphoglossum* | | |
| | | | *Lomagramma* | | |
| | | | *Teratophyllum* | | |
| | *Lomariopsis* | | *Lomariopsis* | 24. Lomariopsidaceae | *Lomariopsis* |
| 21. Marattiaceae | *Marattia* | 16. Marattiaceae | *Marattia* | | |
| 22. Angiopteridaceae | *Angiopteris* | | *Angiopteris* | | |
| | *Archangiopteris* | | | | |
| 23. Marsileaceae | *Marsilea* | 17. Marsileaceae | *Marsilea* | 11. Marsileaceae | *Marsilea* |
| | | 18. Oleandraceae | *Arthropteris* | 25. Tectariaceae | *Arthropteris* |
| | | | *Nephrolepis* | 24. Lomariopsidaceae | *Nephrolepis* |
| | | | *Oleandra* | 26. Oleandraceae | *Oleandra* |
| 24. Ophioglossaceae | *Botrychium* | 19. Ophioglossaceae | *Botrychium* | | |
| | *Helminthostachys* | | *Helminthostachys* | | |
| | *Ophioglossum* | | *Ophioglossum* | | |
| 25. Osmundaceae | *Osmunda* | 20. Osmundaceae | *Osmunda* | 5. Osmundaceae | *Osmunda* |
| 26. Parkeriaceae | *Ceratopteris* | 21. Parkeriaceae | *Ceratopteris* | | |
| 27. Plagiogyriaceae | *Plagiogyria* | 22. Plagiogyriaceae | *Plagiogyria* | 13. Plagiogyriaceae | *Plagiogyria* |
| 28. Polypodiaceae | *Aglaomorpha* | 23. Polypodiaceae | *Aglaomorpha* | 28. Polypodiaceae | *Aglaomorpha* |
| | *Arthromeris* | | *Arthromeris* | | *Arthromeris* |
| | *Belvisia* | | *Belvisia* | | *Belvisia* |

| IFV (1999) | | CPSV (2001) | | Phan (2010) | |
|---|---|---|---|---|---|
| Families | Genera | Families | Genera | Families | Genera |
| | | | | | ***Caobangia*** |
| | *Christiopteris* | | *Christiopteris* | | *Christiopteris* |
| | *Colysis* | | *Colysis* | | |
| | *Crypsinus* | | *Crypsinus* | | |
| | *Drynaria* | | *Drynaria* | | *Drynaria* |
| | *Goniophlebium* | | *Goniophlebium* | | *Goniophlebium* |
| | | | | | *Gymnogrammitis* |
| | | | | | *Kontumia* |
| | | | *Lecanopteris* | | *Lecanopteris* |
| | *Lemmaphyllum* | | *Lemmaphyllum* | | *Lemmaphyllum* |
| | *Lepisorus* | | *Lepisorus* | | *Lepisorus* |
| | *Leptochilus* | | *Leptochilus* | | *Leptochilus* |
| | | | *Loxogramme* | | *Loxogramme* |
| | | | | | *Micropolypodium* |
| | *Microsorum* | | *Microsorum* | | *Microsorum* |
| | *Myrmecopteris* | | | | |
| | *Neocheiropteris* | | *Neocheiropteris* | | *Neocheiropteris* |
| | *Paragramma* | | *Paragramma* | | |
| | *Phymatosorus* | | *Phymatosorus* | | |
| | *Photinopteris* | | | | |
| | *Platycerium* | | *Platycerium* | | *Platycerium* |
| | | | | | ***Polypodioides*** |
| | *Polypodium* | | *Polypodium* | | *Polypodium* |
| | *Pyrrosia* | | *Pyrrosia* | | *Pyrrosia* |
| | *Selliguea* | | *Selliguea* | | *Selliguea* |
| 29. Pteridaceae | *Acrostichum* | 24. Pteridaceae | *Acrostichum* | 18. Pteridaceae | *Acrostichum* |
| | | | | | *Adiantum* |
| | | | | | *Aleuritopteris* |
| | | | | | *Anogramma* |
| | | | | | *Antrophyum* |
| | | | | | *Ceratopteris* |
| | | | | | *Cheilanthus* |
| | | | | | *Coniogramme* |
| | | | | | *Dryopteris* |
| | | | | | *Hemionitis* |
| | | | | | *Mildella* |
| | | | | | *Notholaena* |
| | | | | | *Onychium* |
| | | | | | *Pellaea* |
| | | | | | *Pityrogramma* |
| | *Pteris* | | *Pteris* | | *Pteris* |
| | | | | | *Syngramma* |
| | | | | | *Taenitis* |
| | | | | | *Vittaria* |
| 30. Salviniaceae | *Salvinia* | 25. Salviniaceae | *Salvinia* | 12. Salviniaceae | *Salvinia* |
| | | | | | *Azolla* |
| 31. Schizaeaceae | *Schizaea* | 26. Schizaeaceae | *Schizaea* | 10. Schizaeaceae | *Schizaea* |
| | *Lygodium* | | *Lygodium* | 9. **Lygodiaceae** | *Lygodium* |
| 32. Thelypteridaceae | *Ampelopteris* | 27. Thelypteridaceae | *Ampelopteris* | 20. Thelypteridaceae | |

| **IFV (1999)** | | **CPSV (2001)** | | **Phan (2010)** | |
|---|---|---|---|---|---|
| Families | Genera | Families | Genera | Families | Genera |
| | | | *Amphineuron* | | |
| | *Christella* | | *Christella* | | |
| | *Coryphopteris* | | *Coryphopteris* | | |
| | *Cyclogramma* | | *Cyclogramma* | | |
| | *Cyclosorus* | | *Cyclosorus* | | *Cyclosorus* |
| | | | *Glaphyropteridopsis* | | |
| | | | *Macrothelypteris* | | *Macrothelypteris* |
| | *Metathelypteris* | | *Metathelypteris* | | |
| | | | *Parathelypteris* | | |
| | | | *Phegopteris* | | *Phegopteris* |
| | *Pneumatopteris* | | *Pneumatopteris* | | |
| | *Pronephrium* | | *Pronephrium* | | |
| | *Pseudocyclosorus* | | *Pseudocyclosorus* | | |
| | *Pseudophegopteris* | | *Pseudophegopteris* | | *Pseudophegopteris* |
| | *Sphaerostephanos* | | *Sphaerostephanos* | | |
| | *Stegnogramma* | | *Stegnogramma* | | |
| | *Thelypteris* | | *Thelypteris* | | *Thelypteris* |
| | *Trigonospora* | | *Trigonospora* | | |
| 33. Vittariaceae | *Antrophyum* | 28. Vittariaceae | *Antrophyum* | | |
| | *Vittaria* | | *Vittaria* | | |
| | | 29. Woodsiaceae | *Athyrium* | 21. Woodsiaceae | *Athyrium* |
| | | | | | *Acystopteris* |
| | | | *Cornopteris* | | *Cornopteris* |
| | | | *Cystopteris* | | |
| | | | *Lunathyrium* | | *Deparia* |
| | | | *Diplaziopsis* | | *Diplaziopsis* |
| | | | *Diplazium* | | *Diplazium* |
| | | | *Hypodematium* | | |
| | | | | | *Rhachidosorus* |

**Table S2.** Comparison of classifications of gymnosperm families of Vietnam in IFV and CPSV. (Generic names in boldface were newly recorded for Vietnam).

| **IFV (1999)** | | **CPSV (2001)** | | **Nguyen and Thomas (2005)** | |
|---|---|---|---|---|---|
| **Families** | Genera | Families | Genera | Families | Genera |
| 1. Cycadaceae | *Cycas* | 4. Cycadaceae | as IFV | not recognized | not recognized |
| 2. Gnetaceae | *Gnetum* | 5. Gnetaceae | as IFV | not recognized | not recognized |
| 3. Gingkoaceae | *Gingko* | not recognized | not recognized | not recognized | not recognized |
| 4. Pinaceae | *Abies* | 6. Pinaceae | as IFV | 3. Pinaceae | as IFV |
| | *Keteleeria* | | as IFV | | as IFV |
| | *Pinus* | | as IFV | | as IFV |
| | not recognized | | not recognized | | ***Pseudotsuga*** |
| | *Tsuga* | | as IFV | | as IFV |
| | *Araucaria* | 1. Araucariaceae | *Araucaria* | not recognized | |
| 5. Taxodiaceae | *Cunninghamia* | 9. Taxodiaceae | as IFV | | *Cunninghamia* (in Cupressaceae) |
| | *Glyptostrobus* | | as IFV | not recognized | *Glyptostrobus* (in Cupressaceae) |
| | *Taxodium* | | as IFV | | not recognized |
| | *Cryptomeria* | | not recognized | | not recognized |
| 6. Cupressaceae | *Calocedrus* | 3. Cupressaceae | as IFV | 2. Cupressaceae | as IFV |
| | *Chamaecyparis* | | not recognized | | not recognized |
| | *Cupressus* | | as IFV | | as IFV |
| | *Fokienia* | | as IFV | | as IFV |
| | *Sabina* | | not recognized | | not recognized |
| | not recognized | | *Juniperus* | | not recognized |
| | *Thuja* | | as IFV | | not recognized |
| | not recognized | | not recognized | | ***Taiwania*** |
| | not recognized | | not recognized | | ***Xanthocyparis*** |
| 7. Podocarpaceae | not recognized | 7. Podocarpaceae | *Dacrycarpus* | 4. Podocarpaceae | as CPSV |
| | *Dacrydium* | | as IFV | | as IFV |
| | *Nageia* | | as IFV | | as IFV |
| | *Decussocarpus* | | not recognized | | not recognized |
| | *Podocarpus* | | as IFV | | as IFV |
| 8. Taxaceae | *Taxus* | 8. Taxaceae | as IFV | 6. Taxaceae | as IFV |
| 9. Cephalotaxaceae | *Cephalotaxus* | 2. Cephalotaxaceae | as IFV | 1. Cephalotaxaceae | as IFV |
| 10. Amentotaxaceae | *Amentotaxus* | 9. Taxaceae | as IFV | 5. Taxaceae | as IFV |

**Table S3.** Comparison of classifications of angiosperm families of Vietnam in IFV and CPSV. (IFV families in boldface were newly circumscribed in CPSV).

| IFV (1999–2003) | CPSV (2001–2005) |
|---|---|
| | **MAGNOLIOPSIDA** |
| 1. Magnoliaceae | 1. Magnoliaceae |
| 2. Annonaceae | 2. Annonaceae |
| 3. Myristicaceae | 3. Myristicaceae |
| 4. Chloranthaceae | 6. Chloranthaceae |
| 5. Saururaceae | 9. Saururaceae |
| 6. Piperaceae | 10. Piperaceae |
| 7. Aristolochiaceae | 11. Aristolochiaceae |
| 8. Illiciaceae | 17. Illiciaceae |
| 9. Schisandraceae | 18. Schisandraceae |
| 10. Nelumbonaceae | 19. Nelumbonaceae |
| 11. Nymphaeaceae | 14. Nymphaeaceae |
| 12. Barclayaceae | 15. Barclayaceae |
| 13. Cabombaceae | 13. Cabombaceae |
| 14. Ceratophyllaceae | 16. Ceratophyllaceae |
| 15. Ranunculaceae | 23. Ranunculaceae |
| 16. Berberidaceae | 24. Berberidaceae |
| 17. Sargentodoxaceae | 21. Sargentodoxaceae |
| 18. Lardizabalaceae | 20. Lardizabalaceae |
| 19. Menispermaceae | 22. Menispermaceae |
| 20. Papaveraceae | 25. Papaveraceae |
| 21. Fumariaceae | 26. Fumariaceae |
| 22. Lauraceae | 8. Lauraceae |
| 23. Monimiaceae | 4. Monimiaceae |
| 24. Hernandiaceae | 5. Hernandiaceae |
| 25. Dilleniaceae | 54. Dilleniaceae |
| 26. Actinidiaceae | 81. Actinidiaceae |
| 27. Paeoniaceae | 55. Paeoniaceae |
| 28. Ochnaceae | 56. Ochnaceae |
| 29. Theaceae | 59. Theaceae |
| 30. Dipterocarpaceae | 57. Dipterocarpaceae |
| 31. Ancistrocladaceae | 58. Ancistrocladaceae |
| 32. Stachyuraceae | 66. Stachyuraceaev |
| 33. Pentaphylaceae | 60. Pentaphylaceae |
| 34. Elatinaceae | 64. Elatinaceae |
| 35. Guttiferae | 62. Clusiaceae |
| 36. Elaeocarpaceae | 93. Elaeocarpaceae |
| 37. Tiliaceae | 94. Tiliaceae |
| 38. Sterculiaceae | 95. Sterculiaceae |
| 39. Bombacaceae | 96. Bombacaceae |
| 40. Malvaceae | 97. Malvaceae |
| 41. Nepenthaceae | 118. Nepenthaceae |
| 42. Droseraceae | 117. Droseraceae |
| 43. Flacourtiaceae | 65. Flacourtiaceae |
| 44. Bixaceae | 68. Bixaceae |
| 45. Cochlospermaceae | 69. Cochlospermaceae |
| 46. Violaceae | 67. Violaceae |

| | |
|---|---|
| 47. Tamaricaceae | 79. Tamaricaceae |
| 48. Turneraceae | 71. Turneraceae |
| 49. Passifloraceae | 70. Passifloraceae |
| 50. Caricaceae | 72. Caricaceae |
| 51. Cucurbitaceae | 73. Cucurbitaceae |
| 52. Datiscaeae | 74. Datiscaeae |
| 53. Begoniaceae | 75. Begoniaceae |
| 54. Capparaceae | 76. Capparaceae |
| 55. Brassicaceae | 78. Brassicaceae |
| 56. Moringaceae | 77. Moringaceae |
| 57. Clethraceae | 82. Clethraceae |
| 58. Epacridaceae | 84. Epacridaceae |
| 59. Ericaceae | 83. Ericaceae |
| 60. Sapotaceae | 90. Sapotaceae |
| 61. Ebenaceae | 89. Ebenaceae |
| 62. Styracaceae | 87. Styracaceae |
| 63. Symplocaceae | 88. Symplocaceae |
| 64. Myrsinaceae | 91. Myrsinaceae |
| 65. Primulaceae | 92. Primulaceae |
| 66. Phytolaccaceae | 41. Phytolaccaceae |
| 67. Nyctaginaceae | 42. Nyctaginaceae |
| 68. Aizoaceae | 44. Aizoaceae |
| 69. Cactaceae | 46. Cactaceae |
| 70. Chenopodiaceae | 51. Chenopodiaceae |
| 71. Amaranthaceae | 50. Amaranthaceae |
| 72. Portulacaceae | 47. Portulacaceae |
| 73. Basellaceae | 48. Basellaceae |
| 74. Caryophyllaceae | 49. Caryophyllaceae |
| 75. Polygonaceae | 52. Polygonaceae |
| 76. Plumbaginaceae | 53. Plumbaginaceae |
| 77. Connaraceae | 116. Connaraceae |
| 78. Pittosporaceae | 107. Pittosporaceae |
| 79. Anisophylleaceae | 124. Anisophylleaceae |
| 80. Crassulaceae | 109. Crassulaceae |
| 81. Rosaceae | 111. Rosaceae |
| 82. Suriannaceae | not recognized |
| 83. Saxifragaceae | 110. Saxifragaceae |
| 84. Fabaceae | 115. Fabaceae |
| 85. Elaeagnaceae | 182. Elaeagnaceae |
| 86. Proteaceae | 183. Proteaceae |
| 87. Podostemaceae | 119. Podostemaceae |
| 88. Tritischaceae | not recognized |
| 89. Haloragaceae | 131. Haloragaceae |
| 90. Lecythidaceae | 130. Lecythidaceae |
| 91. Sonneratiaceae | 121. Sonneratiaceae |
| 92. Lythraceae | 120. Lythraceae |
| 93. Crypteroniaceae | 103. Crypteroniaceae |
| 94. Thymelaeaceae | 102.Thymelaeaceae |
| 95. Trapaceae | 129. Trapaceae |
| 96. Myrtaceae | 126. Myrtaceae |
| 97. Punicaceae | 122. Punicaceae |

| | |
|---|---|
| 98. Onagnaceae | 128. Onagnaceae |
| 99. Melastomataceae | 127. Melastomataceae |
| 100. Combretaceae | 125. Combretaceae |
| 101. Rhizophoraceae | 123. Rhizophoraceae |
| 102. Alangiaceae | 157. Alangiaceae |
| 103. Nyssaceae | 154. Nyssaceae |
| 104. Cornaceae | 155. Cornaceae |
| 105. Olacaceae | 172. Olacaceae |
| 106. Opiliaceae | 174. Opiliaceae |
| 107. Santalaceae | 177. Santalaceae |
| 108. Loranthaceae | 179. Loranthaceae |
| 109. Balanophoraceae | 181. Balanophoraceae |
| 110. Celastraceae | 166. Celastraceae |
| 111. Salvadoraceae | 165. Salvadoraceae |
| 112. Aquifoliaceae | 163. Aquifoliaceae |
| 113. Icacinaceae | 164. Icacinaceae |
| 114. Cardiopteridaceae | 176. Cardiopteridaceae |
| 115. Dichapetalaceae | 101. Dichapetalaceae |
| 116. Pandaceae | 100A. Pandaceae |
| 117. Buxaceae | 98. Buxaceae |
| 118. Euphorbiaceae | 100. Euphorbiaceae |
| 119. Oxalidaceae | 148. Oxalidaceae |
| 120. Geraniaceae | 149. Geraniaceae |
| 121. Tropaealaceae | 150. Tropaealaceae |
| 122. Balsaminaceae | 151. Balsaminaceae |
| 123. Erythroxylaceae | 145. Erythroxylaceae |
| 124. Ixonanthaceae | 144. Ixonanthaceae |
| 125. Linaceae | 143. Linaceae |
| 126. Bretschneideraceae | 140. Bretschneideraceae |
| 127. Sapindaceae | 138. Sapindaceae |
| 128. Staphyleaceae | 136. Staphyleaceae |
| 129. Hippocastanaceae | 139. Hippocastanaceae |
| 130. Aceraceae | 137. Aceraceae |
| 131. Sabiaceae | 141. Sabiaceae |
| 132. Malpighiaceae | 146. Malpighiaceae |
| 133. Polygalaceae | 152. Polygalaceae |
| 134. Xanthophyllaceae | not recognized |
| 135. Burseraceae | 132A. Burseraceae |
| 136. Anacardiaceae | 132. Anacardiaceae |
| 137. Simaroubaceae | 133. Simaroubaceae |
| 138. Meliaceae | 135. Meliaceae |
| 139. Rutaceae | 134. Rutaceae |
| 140. Zygophyllaceae | 147. Zygophyllaceae |
| 141. Rhamnaceae | 168. Rhamnaceae |
| 142. Leeaceae | 170. Leeaceae |
| 143. Vitaceae | 169. Vitaceae |
| 144. Apiaceae | 162. Apiaceae |
| 145. Araliaceae | 161. Araliaceae |
| 146. Aralidaceae | 161A. Aralidaceae |
| 147. Platanaceae | 29. Platanaceae |
| 148. Eucomiaceae | 30. Eucomiaceae |

| | |
|---|---|
| 149. Hamamelidaceae | 27. Hamamelidaceae |
| 150. Daphniphyllaceae | 99. Daphniphyllaceae |
| 151. Ulmaceae | 31. Ulmaceae |
| 152. Cannabaceae | 33. Cannabaceae |
| 153. Salicaceae | 80. Salicaceae |
| 154. Moraceae | 32. Moraceae |
| 155. Urticaceae | 34. Urticaceae |
| 156. Rhoipteleaceae | 39. Rhoipteleaceae |
| 157. Juglandaceae | 40. Juglandaceae |
| 158. Myricaceae | 38. Myricaceae |
| 159. Fagaceae | 36. Fagaceae |
| 160. Betulaceae | 37. Betulaceae |
| 161. Casuarinaceae | 35. Casuarinaceae |
| 162. Loganiaceae | 187. Loganiaceae |
| 163. Gentianaceae | 191. Gentianaceae |
| 164. Apocynaceae | 189. Apocynaceae |
| 165. Asclepiadaceae | 190. Asclepiadaceae |
| 166. Solanaceae | 199. Solanaceae |
| 167. Convolvulaceae | 195. Convolvulaceae |
| 168. Cuscutaceae | 196. Cuscutaceae |
| 169. Menyanthaceae | 192. Menyanthaceae |
| 170. Polemoniaceae | 194. Polemoniaceae |
| 171. Hydrophyllaceae | 197. Hydrophyllaceae |
| 172. Boraginaceae | 198. Boraginaceae |
| 173. Verbenaceae | 211. Verbenaceae |
| 174. Lamiaceae | 212. Lamiaceae |
| 175. Callitrichiaceae | 213. Callitrichiaceae |
| 176. Plantaginaceae | 210. Plantaginaceae |
| 177. Buddlejaceae | 200. Buddlejaceae |
| 178. Oleaceae | 171. Oleaceae |
| 179. Scrophulariaceae | 201. Scrophulariaceae |
| 180. Myoporaceae | 208. Myoporaceae |
| 181. Gesneriaceae | 205. Gesneriaceae |
| 182. Acanthaceae | 209. Acanthaceae |
| 183. Pedaliaceae | 203. Pedaliaceae |
| 184. Bignoniaceae | 202. Bignoniaceae |
| 185. Lentibalariaceae | 207. Lentibalariaceae |
| 186. Pentaphragmaceae | not recognized |
| 187. Campanulaceae | 214. Campanulaceae |
| 188. Stylidiaceae | 217. Stylidiaceae |
| 189. Goodeniaceae | 218. Goodeniaceae |
| 190. Rubiaceae | 193. Rubiaceae |
| 191. Carlemanniaceae | not recognized |
| 192. Caprifoliaceae | 184. Caprifoliaceae |
| 193. Valerianaceae | 185. Valerianaceae |
| 194. Dipsacaceae | 186. Dipsacaceae |
| 195. Asteraceae | 219. Asteraceae |
| not recognized | **7. Calycanthaceae** |
| not recognized | 12. Rafflesiaceae |
| not recognized | **28. Altingiaceae** |
| not recognized | **43. Molluginaceae** |

| | |
|---|---|
| not recognized | **45. Tetragoniaceae** |
| not recognized | **61. Bonnetiaceae** |
| not recognized | **63. Hypericaceae** |
| not recognized | **85. Pyrolaceae** |
| not recognized | **86. Diapensiaceae** |
| not recognized | 91A. Theophrastaceae |
| not recognized | **104. Escalloniaceae** |
| not recognized | **105. Iteaceae** |
| not recognized | **106. Hydrangeaceae** |
| not recognized | **108. Penthoraceae** |
| not recognized | **112. Chrysobalanaceae** |
| not recognized | **113. Mimosaceae** |
| not recognized | **114. Ceasalpiniaceae** |
| not recognized | **142. Hugoniaceae** |
| not recognized | **153. Davidiaceae** |
| not recognized | **156. Aucubaceae** |
| not recognized | **158. Mastixiaceae** |
| not recognized | **159. Toricelliaceae** |
| not recognized | **160. Helwingiaceae** |
| not recognized | **167. Siphonodontataceae** |
| not recognized | **173. Schoepfiaceae** |
| not recognized | **175. Erythropalaceae** |
| not recognized | **178. Dipentodontaceae** |
| not recognized | **180. Viscaceae** |
| not recognized | **188. Potaliaceae** |
| not recognized | **204. Martyniaceae** |
| not recognized | **206. Orobanchaceae** |
| not recognized | **215. Lobeliaceae** |
| not recognized | **216. Sphenocleaceae** |
| | **LILIOPSIDA** |
| 196. Limnocharitaceae | 220. Limnocharitaceae |
| 197. Alismataceae | 221. Alismataceae |
| 198. Hydrocharitaceae | 222. Hydrocharitaceae |
| 199. Aponogetonaceae | 223. Aponogetonaceae |
| 200. Potamogetonaceae | 224. Potamogetonaceae |
| 201. Najadaceae | 227. Najadaceae |
| not recognized | **227A. Zosteraceae** |
| 202. Cymodoaceae | 226. Cymodoaceae |
| 203. Triuridaceae | 228. Triuridaceae |
| 204. Pandanaceae | 263. Pandanaceae |
| not recognized | **263A. Freycinetiaceae** |
| 205. Araceae | 261. Araceae |
| not recognized | **261A. Acoraceae** |
| 206. Lemnaceae | 262. Lemnaceae |
| 207. Xyridaceae | 253. Xyridaceae |
| 208. Commelinaceae | 252. Commelinaceae |
| 209. Eriocaulaceae | 254. Eriocaulaceae |
| 210. Flagellariaceae | 257. Flagellariaceae |
| 211. Restionaceae | 255. Restionaceae |
| 212. Centrolepidaceae | 256. Centrolepidaceae |
| 213. Juncaceae | 249. Juncaceae |

| | |
|---|---|
| 214. Typhaceae | 265. Typhaceae |
| 215. Sparganiaceae | 264. Sparganiaceae |
| 216. Arecaceae | 260. Arecaceae |
| 217. Bromeliaceae | 251. Bromeliaceae |
| 218. Musaceae | 241. Musaceae |
| 219. Strelitziaceae | 240. Strelitziaceae |
| 220. Heliconiaceae | 242. Heliconiaceae |
| 221. Lowiaceae | 243. Lowiaceae |
| 222. Zingiberaceae | 245. Zingiberaceae |
| 223. Cannaceae | 246. Cannaceae |
| 224. Marantaceae | 247. Marantaceae |
| 225. Philydraceae | 237. Philydraceae |
| 226. Pontederiaceae | 236. Pontederiaceae |
| 227. Hemodoraceae | not recognized |
| 228. Liliaceae | 229. Liliaceae |
| 229. Smilaceae | 232. Smilaceae |
| 230. Amaryllidaceae | 229.3. Amaryllidaceae |
| 231. Iridaceae | 238. Iridaceae |
| 232. Cyperaceae | 250. Cyperaceae |
| 233. Poaceae | 259. Poaceae |
| 234. Agavaceae | 229.1. Agavaceae |
| 235. Hanguanaceae | 258. Hanguanaceae |
| 236. Taccaceae | 235. Taccaceae |
| 237. Stemonaceae | 233. Stemonaceae |
| 238. Dioscoreaceae | 234. Dioscoreaceae |
| 239. Burmanniaceae | 239. Burmanniaceae |
| 240. Thismiaceae | not recognized |
| 241. Orchidaceae | 248. Orchidaceae |
| not recognized | **225. Ruppiaceae** |
| not recognized | **229.2. Alliaceae** |
| not recognized | **229.4. Asphodelaceae** |
| not recognized | **229.5. Asteliaceae** |
| not recognized | **229.6. Convallariaceae** |
| not recognized | **229.7. Dracaenaceae** |
| not recognized | **229.8. Hemerocallidaceae** |
| not recognized | **229.9. Hyacinthaceae** |
| not recognized | **229.10. Liliaceae s.s** |
| not recognized | **229.11. Melanthiaceae** |
| not recognized | **229.12. Nolinaceae** |
| not recognized | **229.13. Phormiaceae** |
| not recognized | **229.14.Trilliaceae** |
| not recognized | **230. Hypoxidaceae** |
| not recognized | **231. Asparagaceae** |
| not recognized | **244. Costaceae** |

**Table S4.** Classification of lycophytes and ferns of Vietnam according to PPG 1 classification system. (Families in boldface were newly circumscribed for Vietnam).

| PPG I | Lycophytes and Ferns of Vietnam According to PPG 1 | Previous Treatments IFV (1999) | CPSV (2001) | Phan (2010) |
|---|---|---|---|---|
| **LYCOPODIOPSIDA** | | | | |
| **A. Lycopodiales** | | | | |
| 1. Lycopodiaceae | | | | |
| Lycopodielloideae | *Lycopodiella* | not recognized | *Lycopodiella* | Not included |
| Lycopodioideae | *Lycopodium* | *Lycopodium* | as IFV | Not included |
| Huperzioideae | *Huperzia* | *Huperzia* | as IFV | Not included |
| **B. Isoetales** | | | | |
| 2. Isoetaceae | *Isoetes* | *Isoetes* | as IFV | Not included |
| **C. Selaginellales** | | | | |
| 3. Selaginellaceae | *Selaginella* | *Selaginella* | as IFV | Not included |
| **POLYPODIOPSIDA** | | | | |
| **Equisetidae** | | | | |
| **D. Equisetales** | | | | |
| 4. Equisetaceae | *Equisetum* | *Equisetum* | as IFV | as IFV |
| **Ophioglossidae** | | | | |
| **E. Psilotales** | | | | |
| 5. Psilotaceae | *Psilotum* | *Psilotum* | as IFV | as IFV |
| **F. Ophioglossales** | | | | |
| 6. Ophioglossaceae | | | | |
| Helminthostachyoideae | *Helminthostachys* | *Helminthostachys* | as IFV | as IFV |
| Ophioglossoideaa | *Ophioglossum* | *Ophioglossum* | as IFV | as IFV |
| Botrychioideae | *Botrychium* | *Botrychium* | as IFV | as IFV |
| Marattidae | | | | |
| **G. Marattiales** | | | | |
| 7. Marattiaceae | *Angiopteris* (incl. *Archangiopteris*) | *Angiopteris* ( incl. *Archangiopteris*) | as IFV | as IFV |
| | *Christensenia* | | | *Christensenia* |
| | *Marattia* | *Marattia* | as IFV | as IFV |
| **Polypodiidae** | | | | |
| **H. Osmundales** | | | | |
| 8. Osmundaceae | *Osmunda* | *Osmunda* | as IFV | as IFV |
| **I. Hymenophyllales** | | | | |
| 9. Hymenophyllaceae | | | | |
| Trichomanoideae | *Abrodictyum* | | | *Abrodictyum* |
| | *Cephalomanes* | *Cephalomanes* | as IFV | as IFV |
| | *Crepidomanes* | *Crepidomanes* | as IFV | as IFV |
| | *Didymoglossum* | | | *Didymoglossum* |
| | *Trichomanes* | *Trichomanes* | as IFV | as IFV |
| | *Vandenboschia* | *Vandenboschia* | not recognized | as IFV |
| Hymenophylloideae | *Hymenophyllum* | *Hymenophyllum* | as IFV | as IFV |
| **J. Gleicheniales** | | | | |
| 10. Dipteridaceae | *Cheiropleuria* | *Cheiropleuria* (in Cheiropleu-riaceae) | as IFV | *Cheiropleuria* |

| PPG I | Lycophytes and Ferns of Vietnam According to PPG 1 | Previous Treatments IFV (1999) | CPSV (2001) | Phan (2010) |
|---|---|---|---|---|
| | *Dipteris* | *Dipteris* | as IFV | as IFV |
| 11. Gleicheniaceae | *Dicranopteris* | *Dicranopteris* | as IFV | as IFV |
| | *Diplopterygium* | *Diplopterygium* | as IFV | as IFV |
| | *Gleichenia* | *Gleichenia* | as IFV | as IFV |
| **K. Schizaeles** | | | | |
| 12. Lygodiaceae | *Lygodium* | *Lygodium* (in Schizaeaceae) | as IFV | *Lygodium* |
| 13. Schizaeaceae | *Schizaea* | *Schizaea* | as IFV | as IFV |
| **M. Salviniales** | | | | |
| 14. Salviniaceae | *Azolla* | *Azolla* (in Azollaceae) | as IFV | *Azolla* |
| | *Salvinia* | *Salvinia* | as IFV | *Salvinia* |
| 15. Marsileaceae | *Marsilea* | *Marsilea* | as IFV | *Marsilea* |
| **N. Cyatheales** | | | | |
| 16. Plagiogyriaceae | *Plagiogyria* | *Plagiogyria* | as IFV | as IFV |
| 17. Cibotiaceae | *Cibotium* | *Cibotium* (in Thyrsopterida-ceae) | *Cibotium* (in Dicksoniaceae) | *Cibotium* |
| 18. Cyatheaceae | *Alsophila* | not included | as IFV | *Alsophila* |
| | *Cyathea* | *Cyathea* | *Cyathea* | *Cyathea* |
| | *Sphaeropteris* | not recognized | not recognized | *Sphaeropteris* |
| **O. Polypodiales** | | | | |
| Lindsaeineae | | | | |
| 19. Lindsaeaceae | *Lindsaea* | *Lindsaea* (in Dennstaed-tiaceae) | as IFV | *Lindsaea* |
| | *Sphenomeris* | *Sphenomeris* (in Dennstaed-tiaceae) | as IFV | *Sphenomeris* |
| | *Tapeinidium* | *Tapeinidium* (in Dennstaed-tiaceae) | as IFV | *Tapeinidium* |
| Pteridineae | | | | |
| 20. Pteridaceae | | | | |
| Parkerioideae | *Acrostichum* | *Acrostichum* | as IFV | as IFV |
| | *Ceratopteris* | *Ceratopteris* (in Parkeriaceae) | as IFV | *Ceratopteris* |
| Cryptogrammoideae | *Coniogramme* | *Coniogramme* (in Adiantaceae) | as IFV | *Coniogramme* |
| Pteridoideae | *Anogramma* | not recognized | not recognized | *Anogramma* |
| | *Onychium* | *Onychium* (in Adiantaceae) | as IFV | *Onychium* |
| | *Pityrogramma* | *Pityrogramma* (in Adiantaceae) | as IFV | *Pityrogramma* |
| | *Pteris* | *Pteris* | as IFV | as IFV |
| | *Syngramma* | *Syngramma* (in Adiantaceae) | as IFV | *Syngramma* |

| PPG I | Lycophytes and Ferns of Vietnam According to PPG 1 | Previous Treatments IFV (1999) | CPSV (2001) | Phan (2010) |
|---|---|---|---|---|
| | *Taenitis* | *Taenitis* (in Adiantaceae) | as IFV | *Taenitis* |
| Vittarioideae | *Adiantum* | *Adiantum* (in Adiantaceae) | as IFV | *Adiantum* |
| | *Antrophyum* | *Antrophyum* (in Vittariaceae) | as IFV | *Antrophyum* |
| | *Vittaria* | *Vittaria* (in Vittariaceae) | as IFV | *Vittaria* |
| Cheilanthoideae | *Aleuritopteris* | not recognized | not recognized | *Aleuritopteris* |
| | *Hemionitis* | *Hemionitis* (in Adiantaceae) | as IFV | *Hemionitis* |
| | *Mildella* | *Mildella* (in Adiantaceae) | as IFV | *Mildella* |
| | *Notholaena* | *Notholaena* (in Adiantaceae) | as IFV | *Notholaena* |
| 21. Dennstaedtiaceae | *Dennstaedtia* | *Dennstaedtia* | as IFV | as IFV |
| | *Histiopteris* | *Histiopteris* | as IFV | as IFV |
| | *Hypolepis* | *Hypolepis* | as IFV | as IFV |
| | *Microlepia* | *Microlepia* | as IFV | as IFV |
| | *Monachosorium* | *Monachosorium* | as IFV | as IFV |
| | *Pteridium* | *Pteridium* | as IFV | as IFV |
| Aspleniineae | | | | |
| 22. **Cystopteridaceae** | *Acystopteris* | not recognized | not recognized | *Acystopteris* (incl. *Cystopteris*) |
| | *Cystopteris* | *Cystopteris* (in Aspleniaceae) | *Cystopteris* (in Woodsiaceae) | not recognized |
| 23. **Rhachidosoraceae** | *Rhachidosorus* | not recognized | not recognized | *Rhachidosorus* (in Woodsiaceae) |
| 24. **Diplaziopsidaceae** | *Diplaziopsis* | not recognized | *Diplaziopsis* (in Woodsiaceae) | as CPSV |
| 25. Aspleniaceae | *Asplenium* | *Asplenium* | as IFV | as IFV |
| | *Hymenoasplenium* | not recognized | not recognized | *Hymenoasplenium* |
| 26. Blechnaceae | | | | |
| Stenochlaeoideae | *Stenochlaena* | not recognized | *Stenochlaena* | as CPSV |
| Woodwardioideae | *Woodwardi* | not recognized | *Woodwardi* | as CPSV |
| Blechnoideae | *Blechnum* | *Blechnum* | as IFV | as IFV |
| | *Brainea* | *Brainea* | as IFV | as IFV |
| 27. Athyriaceae | *Athyrium* | *Athyrium* (in Aspleniaceae) | *Athyrium* (in Woodsiaceae) | as CPSV |
| | *Deparia* | *Deparia* (incl. *Lunathyrium*) | not recognized | *Deparia* (in Woodsiaceae) |
| | *Diplazium* | *Diplazium* (in Aspleniaceae) | *Diplazium* (in Woodsiaceae) | as CPSV |
| 28. Thelypteridaceae | | | | |
| Phegopteridoideae | *Macrothelypteris* | not recognized | *Macrothelypteris* | as CPSV |
| | *Phegopteris* | not recognized | *Phegopteris* | as CPSV |

| PPG I | Lycophytes and Ferns of Vietnam According to PPG 1 | Previous Treatments IFV (1999) | CPSV (2001) | Phan (2010) |
|---|---|---|---|---|
| | *Pseudophegopteris* | *Pseudophegopteris* | as IFV | as IFV |
| Thelypteridoideae | *Ampelopteris* | *Ampelopteris* | as IFV | not recognized |
| | *Christella* | *Christella* | as IFV | not recognized |
| | *Coryphopteris* | *Coryphopteris* | as IFV | not recognized |
| | *Cyclogramma* | *Cyclogramma* | as IFV | not recognized |
| | *Cyclosorus* | *Cyclosorus* | as IFV | *Cyclosorus* (incl. *Ampelopteris, Christella, Cyclogramma, Glaphyropteridopsis, Pneumatopteris, Pronephrium, Pseudocyclosorus, Stegnogramma, Trigonospora*) |
| | *Glaphyropteridopsis* | not recognized | *Glaphyropteridopsis* | not recognized |
| | *Metathelypteris* | *Metathelypteris* | as IFV | not recognized |
| | *Parathelypteris* | not recognized | *Parathelypteris* | not recognized |
| | *Pneumatopteris* | *Pneumatopteris* | as IFV | not recognized |
| | *Pronephrium* | *Pronephrium* | as IFV | not recognized |
| | *Pseudocyclosorus* | *Pseudocyclosorus* | as IFV | not recognized |
| | *Stegnogramma* | *Stegnogramma* | as IFV | not recognized |
| | *Thelypteris* | *Thelypteris* | as IFV | *Thelypteris* (incl. *Thelypteris, Metathelypteris, Parathelypteris*) |
| | *Trigonospora* | *Trigonospora* | as IFV | not recognized |
| 29. **Didymochlaenaceae** | *Didymochlaena* | *Didymochlaena* (in Dryopteridaceae) | as IFV | as IFV |
| 30. **Hypodematiaceae** | *Hypodematium* | not recognized | *Hypodematium* (in Woodsiaceae) | *Hypodematium* (in Dryopteridaceae) |
| | *Leucostegia* | *Leucostegia* (in Davalliaceae) | as IFV | *Leucostegia* (Dryopteridaceae) |
| 31. Dryopteridaceae | | | | |
| Polybotryoideae | *Polystichopsis* | not recognized | *Polystichopsis* | not recognized |
| Elaphoglosoideae | *Bolbitis* | *Bolbitis* (in Lomariopsidaceae) | as IFV | *Bolbitis* |
| | *Elaphoglossum* | *Elaphoglossum* (in Lomariopsidaceae) | as IFV | *Elaphoglossum* |
| | *Lomagramma* | not recognized | *Lomagramma* (in Lomariopsidaceae) | *Lomagramma* |
| | *Pleocnemia* | not recognized | *Pleocnemia* | *Pleocnemia* |
| | *Rumohra* | *Rumohra* (in Davalliaceae) | as IFV | *Rumohra* |
| | *Teratophyllum* | *Teratophyllum* | as IFV | *Teratophyllum* |

| **PPG I** | **Lycophytes and Ferns of Vietnam According to PPG 1** | **Previous Treatments IFV (1999)** | **CPSV (2001)** | **Phan (2010)** |
|---|---|---|---|---|
| | | (in Lomariopsidaceae) | | |
| Dryopteridoideae | *Arachniodes* | *Arachniodes* | as IFV | *Arachniodes* (incl. *Polystichopsis*) |
| | *Ctenitis* | *Ctenitis* | as IFV | as IFV |
| | *Cyrtomium* | *Cyrtomium* | as IFV | as IFV |
| | not recognized | not recognized | not recognized | *Acrophorus* |
| | not recognized | not recognized | not recognized | *Acrorumohra* |
| | *Dryopteris* (incl. *Acrophorus, Acrorumohra, Dryopsis*) | *Dryopteris* | *Dryopteris* | *Dryopteris* |
| | *Polystichum* | *Polystichum* | as IFV | as IFV |
| 32. **Nephrolepidaceae** | *Nephrolepis* | *Nephrolepis* (in Davalliaceae) | *Nephrolepis* (in Oleandraceae) | *Nephrolepis* (in Lomariopsidaceae) |
| 33. Lomariopsidaceae | *Cyclopeltis* | *Cyclopeltis* (in Dryopteridaceae) | as IFV | *Cyclopeltis* |
| | *Lomariopsis* | *Lomariopsis* | as IFV | as IFV |
| 34. Tectariaceae | *Arthropteris* | *Arthropteris* (in Davalliaceae) | as IFV | *Arthropteris* |
| | *Pteridrys* | *Pteridrys* (in Dryopteridaceae) | as IFV | *Pteridrys* |
| | *Tectaria* | *Tectaria* (in Dryopteridaceae) | as IFV | *Tectaria* |
| 35. Oleandraceae | *Oleandra* | *Oleandra* (in Davalliaceae) | *Oleandra* | as CPSV |
| 36. Davalliaceae | *Davallia* | *Davallia* (incl. *Araiostegia, Davallodes*) | as IFV | as IFV |
| 37. Polypodiaceae | | | | |
| Loxogrammoideae | *Loxogramme* | *Loxogramme* (in Grammitidaceae) | *Loxogramme* | as CPSV |
| Platycerioideae | *Platycerium* | *Platycerium* | as IFV | as IFV |
| | *Pyrrosia* | *Pyrrosia* | as IFV | as IFV |
| Drynarioideae | *Aglaomorpha* | *Aglaomorpha* (incl. *Photinopteris*) | *Aglaomorpha* | as CPSV |
| | *Arthromeris* | *Arthromeris* | as IFV | as IFV |
| | *Gymnogrammitis* | *Gymnogrammitis* (in Davalliaceae) | as IFV | *Gymnogrammitis* |
| | *Selliguea* | *Selliguea* | as IFV | as IFV |
| Microsoroideae | *Goniophlebium* | *Goniophlebium* | as IFV | as IFV |
| | *Lecanopteris* | not recognized | *Lecanopteris* | *Lecanopteris* |
| | not recognized | not recognized | not recognized | *Caobangia* |
| | *Lemmaphyllum* (incl. *Caobangia*) | *Lemmaphyllum* | as IFV | as IFV |
| | *Lepisorus* | *Lepisorus* | as IFV | as IFV |
| | not recognized | not recognized | not recognized | *Kontumia* |

| **PPG I** | **Lycophytes and Ferns of Vietnam According to PPG 1** | **Previous Treatments** | | |
|---|---|---|---|---|
| | | **IFV (1999)** | **CPSV (2001)** | **Phan (2010)** |
| | *Leptochilus* (incl. *Kontumia*) | *Leptochilus* | as IFV | as IFV |
| | *Microsorum* | *Microsorum* | as IFV | as IFV |
| | *Neocheiropteris* | *Neocheiropteris* | as IFV | as IFV |
| | *Paragramma* | *Paragramma* | as IFV | as IFV |
| Polypodioideae | *Polypodium* | *Polypodium* | as IFV | as IFV |
| Grammitidoideae | *Acrosorus* | *Acrosorus* (in Grammitidaceae) | as IFV | *Acrosorus* |
| | *Calymmodon* | *Calymmodon* (in Grammitidaceae) | as IFV | *Calymmodon* |
| | *Cochlidium* | *Cochlidium* (incl. *Xiphopteris*) | as IFV | *Cochlidium* |
| | *Grammitis* | *Grammitis* (in Grammitidaceae) | as IFV | *Grammitis* |
| | *Prosaptia* | *Prosaptia* (in Grammitidaceae) | as IFV | *Prosaptia* |
| | *Scleroglossum* | *Scleroglossum* (in Grammitidaceae) | as IFV | *Scleroglossum* |

**Table S5.** Classification of gymnosperms of Vietnam according to the GPG classification system.

| GPG | Gymnosperms of Vietnam According to "GPG" | IFV (1999) | CPSV (2001) | Nguyen and Thomas (2005) |
|---|---|---|---|---|
| **SUBCLASS I. Cycadidae** Pax **Order A. Cycadales** Pers. ex Bercht. & J.Presl | | | | |
| 1. Cycadaceae | *Cycas* | as GPG | as GPG | not recognized |
| **SUBCLASS II. Ginkgoidae** Engl. **Order B. Ginkgoales** Gorozh. | | | | |
| 2. Ginkgoaceae Engl. | *Ginkgo* | as GPG | not recognized | not recognized |
| **SUBCLASS III. Gnetidae** Pax | | | | |
| **Order C. Gnetales** Blume | | | | |
| 3. Gnetaceae Blume | *Gnetum* | as GPG | as GPG | not recognized |
| **SUBCLASS IV. Pinidae** Cronquist | | | | |
| **Order D. Pinales** Gorozh | | | | |
| 4. Pinaceae Spreng. ex F.Rudolphi, | *Abies* | as GPG | as GPG | as GPG |
| | *Keteleeria* | as GPG | as GPG | as GPG |
| | *Pinus* | as GPG | as GPG | as GPG |
| | *Pseudotsuga* | not recognized | not recognized | as GPG |
| | *Tsuga* | as GPG | as GPG | as GPG |
| **Order E. Araucariales** Gorozh. | | | | |
| 5. Araucariaceae Henkel & W.Hochst. | *Araucaria* | not recognized | as GPG | not recognized |
| 6. Podocarpaceae Endl. | *Dacrycarpus* | not recognized | as GPG | as CPSV |
| | *Dacrydium* | as GPG | as GPG | as GPG |
| | *Nageia* | as GPG | as GPG | as GPG |
| | not recognized | *Decussocarpus* | not recognized | not recognized |
| | *Podocarpus* | as GPG | *Podocarpus* (incl. *Decussocarpus*) | as GPG |
| **Order F. Cupressales** Link | | | | |
| 7. Cupressaceae Gray | *Cunninghamia* | *Cunninghamia* (in Taxodiaceae) | as IFV | as GPG |
| | *Cryptomeria* | *Cryptomeria* (in Taxodiaceae) | not recognized | not recognized |
| | *Glyptostrobus* | *Glyptostrobus* (in Taxodiaceae) | as IFV | as GPG |
| | *Cupressus* (incl. *Xanthocyparis*) | as GPG | as GPG | as GPG |
| | *Fokienia* | as GPG | as GPG | as GPG |
| | *Calocedrus* | as GPG | as GPG | as GPG |
| | *Taiwania* | not recognized | not recognized | as GPG |
| 8. Taxaceae Gray | *Taxus* | *Taxus* | as IFV | as IFV |
| | *Cephalotaxus* | *Cephalotaxus* (in Cephalotaxaceae) | as IFV | as IFV |

| | | | | |
|---|---|---|---|---|
| | *Amentotaxus* | *Amentotaxus* (in Amentotaxaceae) | as GPG | as GPG |

**Table S6.** Comparison of angiosperm families of Vietnam following IFV, CPSV, and APG IV. (Families in **boldface** in IFV and CPSV have the same delimitation; Families in **boldface** in APG IV have the same delimitation as IFV and CPSV).

| APG IV families | IFV (1999-2003) | CPSV (2001-2005) |
|---|---|---|
| Acanthaceae Juss. | **Acanthaceae** | |
| | **Verbenaceae**: *Avincennia* | |
| Achariaceae Harms | Flacourtiaceae: *Hydnocarpus* | Flacourtiaceae: *Gynocardia, Hydnocarpus* |
| Acoraceae Martinov | **Araceae**: *Acorus* | |
| **Actinidiaceae** Gilg & Werderm. | **Actinidiaceae** | |
| Adoxaceae E. Mey. (=Viburnacee Raf.) | **Caprifoliaceae**: *Sambucus, Viburnum* | |
| Aizoaceae Martinov | **Aizoaceae** | |
| | not recognized | Tetragoniaceae |
| Akaniaceae Stapf | **Bretschneideraceae** | |
| Alismataceae Vent. | **Alismataceae** | |
| | Butomaceae: *Tenagocharis, Limnocharis* | Limonocharitaceae: *Tenagocharis, Limnocharis* |
| **Altingiaceae** Lindl. | **Altingiaceae** | |
| Amaranthaceae Juss. | **Amaranthaceae** | |
| | **Chenopodiaceae** | |
| Amaryllidaceae J. St.-Hil. | Amaryllidaceae: *Agapanthus, Crinum, Lycoris, Narcissus, Pancratium, Zephyranthes* | Amaryllidaceae: *Crinum, Lycoris, Narcissus, Pancratium, Zephyranthes* |
| | Liliaceae: *Allium* | Alliaceae |
| **Anacardiaceae** R. Br. | **Anacardiaceae** | |
| **Ancistrocladaceae** Planch. ex Walp. | **Ancistrocladaceae** | |
| **Anisophylleaceae** Ridl. | **Anisophylleaceae** | |
| **Annonaceae** Juss. | **Annonaceae** | |
| Apiaceae Lindl. | **Apiaceae**: excl. *Hydrocotyle* | |
| Apocynaceae Juss. | **Apocynaceae** | |
| | **Asclepiadaceae** | |
| **Aponogetonaceae** Planch. | **Aponogetonaceae** | |
| **Aquifoliaceae** Bercht. & J. Presl | **Aquifoliaceae** | |
| Araceae Juss. | **Araceae**: excl. *Acorus* | |
| | not recognized | Lemnaceae |
| Araliaceae Juss. | **Araliaceae** | |
| | **Apiaceae**: *Hydrocotyle* | |
| **Arecaceae** Bercht. & J. Presl | **Arecaceae** | |
| **Aristolochiaceae** Juss. | **Aristolochiaceae** | |
| Asparagaceae Juss. | **Agavaceae**: *Agave* | |
| | Liliaceae: *Asparagus* | Asparagaceae |
| | Liliaceae: *Aspidistra, Disporopsis, Disporum, Polygonatum, Tupistra* | Convallariaceae |
| | Agavaceae: *Dracaena, Sansevieria* | Dracaenaceae |
| | Agavaceae: *Urginea* | Hyacinthaceae |

| | | |
|---|---|---|
| | Agavaceae: *Nolina* | Nolinaceae: *Nolina* |
| Asphodelaceae Juss. | Liliaceae: *Aloe* | Asphodelaceae |
| | Liliaceae: *Dianella* | Phormiaceae |
| | Liliaceae: *Hemerocallis* | Hemerocallidaceae |
| Asteliaceae Dumort. | not recognized | Asteliaceae |
| **Asteraceae** Bercht. & J. Presl | **Asteraceae** | |
| **Balanophoraceae** Rich. | **Balanophoraceae** | |
| **Balsaminaceae** A. Rich. | **Balsaminaceae** | |
| **Basellaceae** Raf. | **Basellaceae** | |
| **Begoniaceae** C. Agardh | **Begoniaceae** | |
| **Berberidaceae** Juss. | **Berberidaceae** | |
| **Betulaceae** Gray | **Betulaceae** | |
| **Bignoniaceae** Juss. | **Bignoniaceae** | |
| Bixaceae Kunth | **Bixaceae** | |
| | **Cochlospermaceae** | |
| Bonnetiaceae L. Beauvis. ex Nakai | not recognized | Bonnetiaceae |
| **Boraginaceae** Juss. | **Boraginaceae** | |
| **Brassicaceae** Burnett (=Cruciferae Juss.) | **Brassicaceae** | |
| Bromeliaceae Juss. | not recognized | Bromeliaceae |
| **Burmanniaceae** Blume | **Burmanniaceae** | |
| Burseraceae Kunth | Burseraceae | Annacardiaceae: *Bursera, Canarium, Dacryodes, Garuga, Protium* |
| **Buxaceae** Dumort. | **Buxaceae** | |
| **Cabombaceae** Rich ex A. Rich. | **Cabombaceae** | |
| **Cactaceae** Juss. | **Cactaceae** | |
| Calophyllaceae J. Agardh | **Clusiaceae**: *Calophyllym, Mesua* | |
| Calycanthaceae Lindl. | not recognized | Calycanthaceae |
| Campanulaceae Juss. | **Campanulaceae**: excl. *Pentaphragma* | |
| | **Lobeliaceae** | |
| Cannabaceae Martinov | **Cannabaceae** | |
| | **Ulmaceae**: excl. *Ulmus* | |
| **Cannaceae** Juss. | **Cannaceae** | |
| Capparaceae Juss. | **Capparaceae**: excl. *Cleome* | |
| Caprifoliaceae Juss. | Caprifoliaceae: *Lonicera* | Caprifoliaceae: *Abelia, Lonicera* |
| | **Dipsacaceae** | |
| | **Valerianaceae** | |
| Cardiopteridaceae Blume | **Cardiopteridaceae** | |
| | **Icacinaceae**: *Gonocaryum* | |
| **Caricaceae** Dumort. | **Caricaceae** | |
| | **Rubiaceae**: *Carlemannia* | |

| | | |
|---|---|---|
| Carlemanniaceae Airy Shaw | **Caprifoliaceae**: *Silvianthus* | |
| **Caryophyllaceae** Juss. | **Caryophyllaceae** | |
| **Casuarinaceae** R. Br. | **Casuarinaceae** | |
| Celastraceae R. Br. | **Celastraceae** | |
| | not recognized | Siphonodontataceae |
| **Ceratophyllaceae** Gray | **Ceratophyllaceae** | |
| **Chloranthaceae** R. Br. ex Sims | **Chloranthaceae** | |
| Chrysobalanaceae R. Br. | Rosaceae: *Chrysobalanus, Parinari* | Chrysobalanaceae |
| Cleomaceae Bercht. & J. Presl | **Capparaceae**: *Cleome* | |
| **Clethraceae** Klotzsch | **Clethraceae** | |
| Clusiaceae Lindl. | **Clusiaceae**: *Garcinia* | |
| Colchicaceae DC. | **Liliaceae**: *Gloriosa, Iphigenia* | |
| | **Liliaceae**: *Disporum* | |
| **Combretaceae** R. Br. | **Combretaceae** | |
| **Commelinaceae** Mirb. | **Commelinaceae** | |
| **Connaraceae** R. Br. | **Connaraceae** | |
| Convolvulaceae Juss. | **Convolvulaceae** | |
| | not recognized | Cuscutaceae |
| Cornaceae Bercht. & J. Presl | Cornaceae: *Cornus* | Cornaceae |
| | **Alangiaceae** | |
| Costaceae Nakai | Zingiberaceae: *Costus* | Costaceae: *Costus* |
| **Crassulaceae** J.St.-Hil. | **Crassulaceae** | |
| **Crypteroniaceae** A. DC. | **Crypteroniaceae** | |
| **Cucurbitaceae** Juss. | **Cucurbitaceae** | |
| Cymodoceaceae Vines | Zannichelliaceae | Cymodoceaceae |
| **Cyperaceae** Juss. | **Cyperaceae** | |
| **Daphniphyllaceae** Mull. Arg. | **Daphniphyllaceae** | |
| **Datiscaceae** Dumort. | **Datiscaceae** | |
| Diapensiaceae Lindl. | not recognized | Diapensiaceae |
| **Dichapetalaceae** Baill. | **Dichapetalaceae** | |
| **Dilleniaceae** Salisb. | **Dilleniaceae** | |
| Dioscoreaceae R. Br. | **Dioscoreaceae** | |
| | **Taccaceae** | |
| Dipentodontaceae Merr. | not recognized | Dipentodontaceae |
| **Dipterocarpaceae** Blume | **Dipterocarpaceae** | |
| **Droseraceae** Salisb. | **Droseraceae** | |

| | | |
|---|---|---|
| **Ebenaceae** Gurke | **Ebenaceae** | |
| **Elaeagnaceae** Juss. | **Elaeagnaceae** | |
| **Elaeocarpaceae** Juss. | **Elaeocarpaceae** | |
| **Elatinaceae** Dumort. | **Elatinaceae** | |
| Ericaceae Juss. | **Ericaceae** | |
| | **Epacridaceae** | |
| | not recognized | Pyrolaceae |
| **Eriocaulaceae** Martinov | **Eriocaulaceae** | |
| **Erythroxylaceae** Kunth | **Erythroxylaceae** | |
| Escallonoiaceae R. Br. ex Dumort. | Saxifragaceae: *Polyosma* | Escallonoiaceae: *Polyosma* |
| **Eucommiaceae** Engl. | **Eucommiaceae** | |
| Euphorbiaceae Juss. | Euphorbiaceae: *Acalypha, Alchornea, Aleurites, Baliospermum, Blachia, Chaetocarpus, Cladogynos, Claoxylon, Cleidiocarpon, Cleidion, Cnesmone, Codiaeum, Croton, Dalechampia, Duetzianthus, Dimorphocalyx, Endospermum, Epiperinus, Erismanthus, Euphorbia, Excoecaria, Hevea, Homonoia, Hura, Jatropha, Lasiococca, Macaranga, Mallotus, Manihot, Melanolepis, Ostodes, Pachystylidium, Pedilanthus, Ricinus, Strophioblachia, Sumbaviopsis, Suregada, Trevia, Trigonostemon, Vernicia*. | Euphorbiaceae: *Acalypha, Alchornea, Aleurites, Baliospermum, Blachia, Chaetocarpus, Cladogynos, Claoxylon, Cleidiocarpon, Cleidion, Cnesmone, Codiaeum, Croton, Dalechampia, Duetzianthus, Dimorphocalyx, Endospermum, Epiperinus, Erismanthus, Euphorbia, Excoecaria, Hevea, Homonoia, Hura, Jatropha, Koilodepas, Lasiococca, Macaranga, Mallotus, Manihot, Melanolepis, Mercurialis, Ostodes, Pachystylidium, Pedilanthus, Ricinus, Strophioblachia, Sumbaviopsis, Suregada, Trevia, Trigonostemon, Vernicia*. |
| Fabaceae Lindl. | **Ceasalpiniaceae** | |
| | **Fabaceae** | |
| | **Ceasalpiniaceae** | |
| **Fagaceae** Dumort. | **Fagaceae** | |
| **Flagellariaceae** Dumort. | **Flagellariaceae** | |
| Garryaceae Lindl. | Cornaceae: *Aucuba* | Aucubaceae |
| Gentianaceae Juss. | **Gentianaceae** | |
| | **Potaliaceae** | |
| **Geraniaceae** Juss. | **Geraniaceae** | |
| **Gesneriaceae** Rich. & Juss. | **Gesneriaceae** | |
| Gisekiaceae Nakai | Aizoaceae: *Gisekia* | Molluginaceae: *Gisekia* |
| **Goodeniaceae** R. Br. ex Rich. | **Goodeniaceae** | |
| **Haloragaceae** R. Br. | **Haloragaceae** | |
| **Hamamelidaceae** R. Br. | **Hamamelidaceae** | |
| **Hanguanaceae** Airy Shaw | **Hanguanaceae** | |
| **Heliconiaceae** Vines | **Heliconiaceae** | |
| Helwingiaceae Decne. | Cornaceae: *Helwingia* | Helwingiaceae |

| | | |
|---|---|---|
| **Hernandiaceae** Blume | **Hernandiaceae** | |
| **Hydrangeaceae** Dumort. | **Hydrangeaceae** | |
| Hydrocharitaceae Juss. | **Hydrocharitaceae** | |
| | **Najadaceae** | |
| **Hydroleaceae** R. Br. | **Hydroleaceae** | |
| Hypericaceae Juss. | Clusiaceae: *Cratoxylum, Hypericum* | Hypericaceae |
| Hypoxidaceae R. Br. | Amaryllidaceae: *Curculigo, Hypoxis* | Hypoxidaceae |
| Icacinaceae Miers | **Icacinaceae**: excl. *Gomphandra, Gonocaryum* | |
| **Iridaceae** Juss. | **Iridaceae** | |
| Iteaceae J. Agardh | Saxifragaceae: *Itea* | Iteaceae |
| **Ixonanthaceae** Planch. ex Miq. | **Ixonanthaceae** | |
| Juglandaceae DC. Ex Perleb | **Juglandaceae** | |
| | **Rhoipteleaceae** | |
| **Juncaceae** Juss. | **Juncaceae** | |
| Lamiaceae Martinov | **Lamiaceae** | |
| | **Verbenaceae**: *Callicarpa, Caryopteris, Clerodendron, Congea, Garrettia, Gmelina, Hymenopyramis, Karomia, Prema, Schnabelia, Sphenodesme, Tectona, Teijsmanniodendron, Tsoongia, Vitex* | |
| **Lardizabalaceae** R. Br. | **Lardizabalaceae** | |
| | **Sargentodoxaceae** | |
| **Lauraceae** Juss. | **Lauraceae** | |
| **Lecythidaceae** A. Rich. | **Lecythidaceae** | |
| **Lentibulariaceae** Rich. | **Lentibulariaceae** | |
| Liliaceae Juss. | Liliaceae: *Lilium* | Liliaceae s.s. |
| Linaceae DC. ex Perleb. | **Linaceae** | |
| | Sabiaceae: *Hugonia, Indoroucheria* | Hugoniaceae |
| Linderniaceae Borsch et al. | **Scrophulariaceae**: *Legazpia, Linderna, Picria, Pierranthus, Torenia* | |
| Loganiaceae R. Br. ex Mart. | **Loganiaceae**: *Mitrasacme, Mitreola, Strychnos* | |
| Loranthaceae Juss. | **Loranthaceae** | |
| | Saxifragaceae: *Pilostigma* | |
| **Lowiaceae** Ridl. | **Lowiaceae** | |
| Lythraceae J. St.-Hil. | **Lythraceae** | |
| | **Punicaceae** | |
| | **Sonneratiaceae** | |
| | **Trapaceae** | |
| **Magnoliaceae** Juss. | **Magnoliaceae** | |
| **Malpighiaceae** Juss. | **Malpighiaceae** | |
| Malvaceae Juss. | **Bombacaceae** | |
| | **Malvaceae** | |
| | **Sterculiaceae** | |
| | **Tiliaceae** | |

| | | |
|---|---|---|
| **Marantaceae** R. Br. | **Marantaceae** | |
| **Martyniaceae** Horan. | **Martyniaceae** | |
| Mazaceae Reveal | **Scrophulariaceae**: *Mazus* | |
| Melanthiaceae Batsch ex Borkh. | Liliaceae: *Paris* | Trilliaceae |
| **Melastomataceae** Juss. | **Melastomataceae** | |
| **Meliaceae** Juss. | **Meliaceae** | |
| **Menispermaceae** Juss. | **Menispermaceae** | |
| **Menyanthaceae** Dumort. | **Menyanthaceae** | |
| Mitrastemonaceae Makino | **Rafflesiaceae**: *Mitrastemon* | |
| Molluginaceae Bartl. | **Molluginaceae**: *Glinus, Mollugo* | |
| **Monimiaceae** Juss. | **Monimiaceae** | |
| **Moraceae** Gaudich. | **Moraceae** | |
| **Moringaceae** Martinov | **Moringaceae** | |
| **Musaceae** Juss. | **Musaceae** | |
| **Myricaceae** Rich. ex Kunth | **Myricaceae** | |
| **Myristicaceae** R. Br. | **Myristicaceae** | |
| **Myrtaceae** Juss. | **Myrtaceae** | |
| **Nelumbonaceae** A. Rich. | **Nelumbonaceae** | |
| **Nepenthaceae** Dumort | **Nepenthaceae** | |
| **Nyctaginaceae** Juss. | **Nyctaginaceae** | |
| Nymphaeaceae Salisb. | **Nymphaeaceae** | |
| | **Barclayaceae** | |
| Nyssaceae Juss. ex Dumort. | Cornaceae: *Mastixia* | Mastixiaceae |
| | **Nyssaceae** | |
| | not recognized | Davidiaceae |
| **Ochnaceae** DC. | **Ochnaceae** | |
| Olacaceae R. Br. | **Olacaceae** | |
| | Oleaceae: *Erythropalum* | Erythropalaceae |
| Oleaceae Hoffmanns. & Link | Oleaceae: excl. *Erythropalum* | Oleaceae |
| **Opiliaceae** Veleton | **Opiliaceae** | |
| **Orchidaceae** Juss. | **Orchidaceae** | |
| Orobanchaceae Vent. | **Orobanchaceae** | |
| | Scrophulariaceae: *Alectra, Brandisia, Centranthera, Lindendergia, Pedicularis, Rehmannia, Sopubia, Wightia* | Scrophulariaceae: *Alectra, Brandisia, Buchnera, Centranthera, Lindendergia, Pedicularis, Rehmannia, Sopubia, Striga, Wightia* |
| **Oxalidaceae** R. Br. | **Oxalidaceae** | |
| **Paeoniaceae** Raf. | **Paeoniaceae** | |
| **Pandanaceae** R. Br., | **Pandanaceae** | |
| Papaveraceae Juss. | **Papaveraceae** | |
| | **Fumariaceae** | |

| | | |
|---|---|---|
| Passifloraceae Juss. ex Roussel | **Passifloraceae** | |
| | **Turneraceae** | |
| Paulowniaceae Nakai | **Scrophulariaceae**: *Paulownia* | |
| **Pedaliaceae** R. Br. | **Pedaliaceae** | |
| Pentaphragmataceae J. Agardh | **Campanulaceae**: *Pentaphragma* | |
| Pentaphylacaceae Engl. | **Pentaphylacaceae** | |
| | Theaceae: *Adinandra, Anneslea, Eurya, Ternstroemia* | Theaceae: *Adinandra, Anneslea, Eurya* |
| Penthoraceae Rydb. ex Britton | Pittosporaceae: *Penthorum* | Penthoraceae |
| Petrosaviaceae Hutch. | Liliaceae: *Petrosavia* | Melanthiaceae: *Petrosavia* |
| **Philydraceae** Link | **Philydraceae** | |
| Phrymaceae Schauer | Scrophulariaceae: *Mimulus, Mazus, Microcarpaea* | Scrophulariaceae: *Mimulus, Microcarpaea, Glossotigma* |
| Phyllanthaceae Martinov | **Euphorbiaceae**: *Aporosa, Actephila, Antidesma, Aporosa, Baccaurea, Bischofia, Breynia, Bridelia, Cleistanthus, Flueggea, Glochidion, Hymenocardia, Leptopus, Margaritaria, Phyllanthus, Sauropus* | |
| **Phytolaccaceae** R. Br. | **Phytolaccaceae** | |
| Piperaceae Giseke | Piperaceae | Piperaceae: excl. *Circaeocarpus* |
| Pittosporaceae R. Br. | Pittosporaceae: excl. *Penthorum* | Pittosporaceae |
| Plantaginaceae Juss. | **Plantaginaceae** | |
| | Scrophulariaceae: *Angelonia* | Scrophulariaceae: *Adenosma, Angelonia, Antirrhinum, Bacopa, Digitalis, Limnophila, Russelia, Scoparia, Stemodia, Veronica* |
| | **Callitrichiaceae** | |
| **Platanaceae** T. Lestib. | **Platanaceae** | |
| **Plumbaginaceae** Juss. | **Plumbaginaceae** | |
| **Poaceae** Barnhart | **Poaceae** | |
| **Podostemaceae** Rich. ex Kunth | **Podostemaceae** | |
| **Polemoniaceae** Juss. | **Polemoniaceae** | |
| **Polygalaceae** Hoffmanns. & Link | **Polygalaceae** | |
| **Polygonaceae** Juss. | **Polygonaceae** | |
| **Pontederidaceae** Kunth. | **Pontederidaceae** | |
| Portulacaceae Juss. | **Portulacaceae:** excl. *Talinum* | |
| **Potamogetonaceae** Bercht. & J. Presl. | **Potamogetonaceae** | |
| Primulaceae Batsch ex Borkh. | **Myrsinaceae** | |
| | **Primulaceae** | |
| **Proteaceae** Juss. | **Proteaceae** | |
| Putranjivaceae Meisn. | **Euphorbiaceae**: *Drypetes* | |
| Rafflesiaceae Dumort. | **Rafflesiaceae**: *Sapria* | |

| | | |
|---|---|---|
| **Ranunculaceae** Juss. | **Ranunculaceae** | |
| Resedaceae Martinov | Capparaceae: *Neothorelia, Stixis, Tirania* | Capparaceae: *Stixis, Tirania* |
| Restionaceae R. Br. | **Restionaceae** | |
| Restionaceae R. Br. | **Centrolepidaceae** | |
| **Rhamnaceae** Juss. | **Rhamnaceae** | |
| **Rhizophoraceae** Pers. | **Rhizophoraceae** | |
| Rosaceae Juss. | Rosaceae: excl. *Chrysobalanus, Parinari* | Rosaceae |
| Rubiaceae Juss. | Rubiaceae: excl. *Carlemannia* | |
| **Ruppiaceae** Horan. | **Ruppiaceae** | |
| Rutaceae Juss. | **Rutaceae** | |
| Rutaceae Juss. | **Simaroubaceae**: *Harrisonia* | |
| Sabiaceae Blume | Sabiaceae: excl. *Hugonia, Indoroucheria* | Sabiaceae |
| Salicaceae Mirb. | Flacourtiaceae: excl. *Hydnocarpus* | Flacourtiaceae: excl. *Gynocardia, Hydnocarpus* |
| Salicaceae Mirb. | **Salicaceae** | |
| **Salvadoraceae** Lindl | **Salvadoraceae** | |
| Santalaceae R. Br. | **Santalaceae** | |
| Santalaceae R. Br. | Loranthaceae: *Ginalloa, Korthalsella, Viscum* | Viscaceae |
| Sapindaceae Juss. | **Aceraceae** | |
| Sapindaceae Juss. | **Hippocastanaceae** | |
| Sapindaceae Juss. | **Sapindaceae** | |
| **Sapotaceae** Juss. | **Sapotaceae** | |
| **Saururaceae** Rich. ex T. Lestib. | **Saururaceae** | |
| Saxifragaceae Juss. | Saxifragaceae: excl. *Pilostigma* | Saxifragaceae |
| Schisandraceae Blume | **Schisandraceae** | |
| Schisandraceae Blume | **Illiciaceae** | |
| Schoepfiaceae Blume | Olacaceae: *Schoepfia* | Schoepfiaceae |
| Scrophulariaceae Juss. | **Buddlejaceae** | |
| Scrophulariaceae Juss. | **Myoporaceae** | |
| Scrophulariaceae Juss. | **Scrophulariaceae**: *Scrophularia* | |
| Simaroubaceae DC. | **Simaroubaceae**: excl. *Irvingia* | |
| **Smilacaceae** Vent. | **Smilacaceae** | |
| **Solanaceae** Juss. | **Solanaceae** | |
| **Sphenocleaceae** T. Baskerv. | **Sphenocleaceae** | |
| **Stachyuraceae** J. Agardh | **Stachyuraceae** | |
| Staphyleaceae Martinov | **Staphyleaceae**: excl. *Tapiscia* | |
| **Stemonaceae** Caruel | **Stemonaceae** | |
| Stemonuraceae Karehed | **Icacinaceae**: *Gomphandra* | |
| **Strelitziaceae** Hutch. | **Strelitziaceae** | |
| **Stylidiaceae** R. Br. | **Stylidiaceae** | |
| **Styracaceae** DC. & Spreng. | **Styracaceae** | |
| Surianaceae Arn. | not recognized | Simaroubaceae: *Suriana* |
| **Symplocaceae** Desf. | **Symplocaceae** | |
| Talinaceae Doweld | **Portulacaceae**: *Talinum* | |

| | | |
|---|---|---|
| **Tamaricaceae** Link | **Tamaricaceae** | |
| Tapisciaceae Takht. | **Staphyleaceae**: *Tapiscia* | |
| Theaceae Mirb. | Theaceae: excl. *Adinandra, Anneslea, Eurya, Temstroemia* | Theaceae: excl. *Adinandra, Anneslea, Eurya* |
| **Thymelaeaceae** Juss. | **Thymelaeaceae** | |
| Torricelliaceae Hu | Cornaceae: *Toricellia* | Toricelliaceae |
| **Triuridaceae** Gardner | **Triuridaceae** | |
| **Tropaeolaceae** Juss. ex DC. | **Tropaeolaceae** | |
| Typhaceae Juss. | **Sparganiaceae** | |
| | **Typhaceae** | |
| Ulmaceae Mirb. | **Ulmaceae**: *Ulmus* | |
| **Urticaceae** Juss. | **Urticaceae** | |
| Verbenaceae J. St. Hil. | **Verbenaceae**: *Duranta, Lantana, Phyla, Stachtarpheta, Verbena* | |
| **Violaceae** Batsch | **Violaceae** | |
| Vitaceae Juss. | **Vitaceae** | |
| | **Leeaceae** | |
| **Xyridaceae** C. Agardh | **Xyridaceae** | |
| Costaceae Nakai | Zingiberaceae: *Costus* | Costaceae: *Costus* |
| Zingiberaceae Martinov | Zingiberaceae: excl. *Costus* | Zingiberaceae |
| Zosteraceae Dumort. | not recognized | Zosteraceae |
| **Zygophyllaceae** R. Br. | **Zygophyllaceae** | |

**Table S7.** Classification of angiosperm families in Vietnam according to the APG IV classifications. (Families in **boldface** in IFV and CPSV have the same delimitation; Families in **boldface** in APG IV have the same delimitation as IFV and CPSV).

| APG IV families | IFV (1999-2003) | CPSV (2001-2005) |
|---|---|---|
| **BASAL ANGIOSPERMS** | | |
| **Nymphaeales** Salisb. ex Bercht. & J. Presl | | |
| 1 [3]. **Cabombaceae** Rich ex A. Rich. | as APG IV | as APG IV |
| 2 [4]. Nymphaeaceae Salisb. | **Nymphaeaceae**<br>**Barclayaceae** | as IFV<br>as IFV |
| **Austrobaileyales** Takht. ex Reveal | | |
| 3 [7]. Schisandraceae Blume | **Schisandraceae**<br>**Illiciaceae** | as IFV<br>as IFV |
| **MAGNOLIIDS** | | |
| **Piperales** Bercht. & J. Presel | | |
| 4 [10]. **Saururaceae** Rich. ex T. Lestib. | as APG IV | as APG IV |
| 5 [12]. Piperaceae Giseke | Piperaceae | Piperaceae: excl. *Circaeocarpus* |
| 6 [12]. **Aristolochiaceae** Juss. | as APG IV | as APG IV |
| **Magnoliales** Juss. ex Bercht. & J. Presl | | |
| 7 [113]. **Myristicaceae** R. Br. | as APG IV | as APG IV |
| 8 [14]. **Magnoliaceae** Juss. | as APG IV | as APG IV |
| 9 [18]. **Annonaceae** Juss. | as APG IV | as APG IV |
| **Laurales** Juss. ex Bercht. & J. Presl | | |
| 10 [19]. Calycanthaceae Lindl. | not recognized | Calycanthaceae |
| 11 [23]. **Hernandiaceae** Blume | as APG IV | as APG IV |
| 12 [24]. **Monimiaceae** Juss. | as APG IV | as APG IV |
| 13 [25]. **Lauraceae** Juss. | as APG IV | as APG IV |
| **INDEPENDENT LINEAGE: UNPLACED TO MORE INCLUSIVE CLADE** | | |
| **Chloranthales** Mart. | | |
| 14 [26]. **Chloranthaceae** R. Br. ex Sims | as APG IV | as APG IV |
| **MONOCOTS** | | |
| **Acorales** Mart. | | |
| 15 [27]. Acoraceae Martinov | **Araceae**: *Acorus* | as APG IV |
| **Alismatales** R. Br. ex Bercht. & J. Presl | | |
| 16 [28]. Araceae Juss. | **Araceae**: excl. *Acorus* | as APG IV |
| 17 [30]. Alismataceae Vent. | **Alismataceae**<br>**Butomaceae**: *Tenagocharis, Limnocharis* | as IFV |
| 18 [32]. Hydrocharitaceae Juss. | **Hydrocharitaceae**<br>**Najadaceae** | as IFV<br>as IFV |
| 19 [34]. **Aponogetonaceae** Planch. | as APG IV | as APG IV |
| 20 [37]. Zosteraceae Dumort. | not recognized | as APG IV |
| 21 [38]. **Potamogetonaceae** Bercht. & J. Presl. | as APG IV | as APG IV |
| 22 [40]. **Ruppiaceae** Horan. | as APG IV | as APG IV |
| 23 [41]. Cymodoceaceae Vines | Zannichelliaceae | Cymodoceaceae |
| **Petrosaviales** Hutch. | | |
| 24 [42]. Petrosaviaceae Hutch. | Liliaceae: *Petrosavia* | Melanthiaceae: *Petrosavia* |
| **Dioscoreales** Mart. | | |
| 25 [44]. **Burmanniaceae** Blume | as APG IV | as APG IV |
| 26 [45]. Dioscoreaceae R. Br. | **Dioscoreaceae** | as APG IV |

| | | |
|---|---|---|
| | **Taccaceae** | as APG IV |
| **Pandanales** R. Br. ex Bercht. & J. Presl | | |
| 27 [46]. **Triuridaceae** Gardner | as APG IV | as APG IV |
| 28 [48]. **Stemonaceae** Caruel | as APG IV | as APG IV |
| 29 [50]. **Pandanaceae** R. Br. | as APG IV | as APG IV |
| **Liliales** Perleb | | |
| 30 [53]. Melanthiaceae Batsch ex Borkh. | Liliaceae: *Paris* | Trilliaceae |
| 31 [56]. Colchicaceae DC. | Liliaceae: *Gloriosa, Iphigenia*<br>Liliaceae: *Disporum* | Melanthiaceae: *Gloriosa, Iphigenia*<br>Convallariaceae: *Disporum* |
| 32 [59]. **Smilacaceae** Vent. | as APG IV | as APG IV |
| 34 [60]. Liliaceae Juss. | Liliaceae: *Lilium* | Liliaceae |
| **Asparagales Link** | | |
| 35 [61]. **Orchidaceae** Juss. | as APG IV | as APG IV |
| 36 [64]. Asteliaceae Dumort. | not recognized | Asteliaceae |
| 37 [66]. Hypoxidaceae R. Br. | Amaryllidaceae: *Curculigo, Hypoxis* | Hypoxidaceae |
| 38 [70]. **Iridaceae** Juss. | as APG IV | as APG IV |
| 39 [72]. Asphodelaceae Juss. | Liliaceae: *Aloe*<br>Liliaceae: *Dianella*<br>Liliaceae: *Hemerocallis* | Asphodelaceae<br>Phormiaceae<br>Hemerocallidaceae |
| 40 [73]. Amaryllidaceae J. St.-Hil. | Amaryllidaceae: *Agapanthus, Crinum, Lycoris, Narcissus, Pancratium, Zephyranthes*<br>Liliaceae: *Allium* | Amaryllidaceae: *Crinum, Lycoris, Narcissus, Pancratium, Zephyranthes*<br>Alliaceae |
| 41. [74]. Asparagaceae Juss. | **Agavaceae**: *Agave*<br>Liliaceae: *Asparagus*<br>Liliaceae: *Aspidistra, Disporopsis, Disporum, Polygonatum, Tupistra*<br>Agavaceae: *Dracaena, Sansevieria*<br>Agavaceae: *Urginea*<br>Agavaceae: *Nolina* | Asparagaceae<br>Convallariaceae: excl. *Disporum*<br>Dracaenaceae<br>Hyacinthaceae<br>Nolinaceae: *Nolina* |
| **Arecales Bromhead** | | |
| 42 [76]. **Arecaceae** Bercht. & J. Presl | as APG IV | as APG IV |
| **Commelinales** Mirb. ex Bercht. J. Presl | | |
| 43 [77]. **Hanguanaceae** Airy Shaw | as APG IV | as APG IV |
| 44 [78]. **Commelinaceae** Mirb. | as APG IV | as APG IV |
| 45 [79]. **Philydraceae** Link | as APG IV | as APG IV |
| 46 [80]. **Pontederidaceae** Kunth. | as APG IV | as APG IV |
| **Zingiberales** Griseb. | | |
| 47 [82]. **Strelitziaceae** Hutch. | as APG IV | as APG IV |
| 48 [83]. **Lowiaceae** Ridl. | as APG IV | as APG IV |
| 49 [84]. **Heliconiaceae** Vines | as APG IV | as APG IV |
| 50 [85]. **Musaceae** Juss. | as APG IV | as APG IV |
| 51 [86]. **Cannaceae** Juss. | as APG IV | as APG IV |
| 52 [87]. **Marantaceae** R. Br. | as APG IV | as APG IV |
| 53 [88]. Costaceae Nakai | Zingiberaceae: *Costus* | Costaceae: *Costus* |

| | | |
|---|---|---|
| 54 [89]. Zingiberaceae Martinov | Zingiberaceae: excl. *Costus* | Zingiberaceae |
| **Poales** Small | | |
| 55 [90]. Typhaceae Juss. | **Sparganiaceae**<br>**Typhaceae** | as IFV<br>as IFV |
| 56 [91]. **Bromeliaceae** Juss. | as APG IV | as APG IV |
| 57 [93]. **Xyridaceae** C. Agardh | as APG IV | as APG IV |
| 58 [94]. **Eriocaulaceae** Martinov | as APG IV | as APG IV |
| 59 [97]. **Juncaceae** Juss. | as APG IV | as APG IV |
| 60 [98]. **Cyperaceae** Juss. | as APG IV | as APG IV |
| 61 [99]. Restionaceae R. Br. | **Restionaceae**<br>**Centrolepidaceae** | as IFV<br>as IFV |
| 62 [100]. **Flagellariaceae** Dumort. | as APG IV | as APG IV |
| 63 [103]. **Poaceae** Barnhart | as APG IV | as APG IV |
| **PROBABLE SISTER OF EUDICOTS** | | |
| **Ceratophyllales** Link | | |
| 64 [104]. **Ceratophyllaceae** Gray | as APG IV | as APG IV |
| **EUDICOTS** | | |
| **Ranunculales** Juss. ex Bercht. & J. Presl | | |
| 65 [106]. Papaveraceae Juss. | **Papaveraceae**<br>**Fumariaceae** | as IFV<br>as IFV |
| 66 [108]. Lardizabalaceae R. Br. | **Lardizabalaceae**<br>**Sargentodoxaceae** | as IFV<br>as IFV |
| 67 [109]. **Menispermaceae** Juss. | as APG IV | as APG IV |
| 68 [110]. **Berberidaceae** Juss. | as APG IV | as APG IV |
| 69 [111]. **Ranunculaceae** Juss. | as APG IV | as APG IV |
| **Proteales** Juss. ex Bercht. & J. Presl | | |
| 70 [112]. Sabiaceae Blume | Sabiaceae: excl. *Hugonia, Indoroucheria* | as APG IV |
| 71 [113]. **Nelumbonaceae** A. Rich. | as APG IV | as APG IV |
| 72 [114]. **Platanaceae** T. Lestib. | as APG IV | as APG IV |
| 73 [115]. **Proteaceae** Juss. | as APG IV | as APG IV |
| **Buxales** Takht. ex Reveal | | |
| 74 [117]. **Buxaceae** Dumort. | as APG IV | as APG IV |
| **CORE EUDICOTS** | | |
| #**Dilleniales** DC. ex Bercht. & J. Presl | | |
| 75 [120]. **Dilleniaceae** Salisb. | as APG IV | as APG IV |
| **SUPERROSIDS** | | |
| **Saxifragales** Bercht. & J. Presl. | | |
| 76 [122]. **Paeoniaceae** Raf. | as APG IV | as APG IV |
| 77 [123]. **Altingiaceae** Lindl. | as APG IV | as APG IV |
| 78 [124]. **Hamamelidaceae** R. Br. | as APG IV | as APG IV |
| 79 [127]. Iteaceae J. Agardh | Saxifragaceae: *Itea* | Iteaceae |
| 80 [129]. Saxifragaceae Juss. | Saxifragaceae: excl. *Pilostigma* | Saxifragaceae |
| 81 [130]. **Crassulaceae** J.St.-Hil. | as APG IV | as APG IV |
| 82 [133]. Penthoraceae Rydb. ex Britton | Pittosporaceae: *Penthorum* | Penthoraceae |
| 83 [134]. **Haloragaceae** R. Br. | as APG IV | as APG IV |

| | | |
|---|---|---|
| **ROSIDS** | | |
| **Vitales** Juss. ex Bercht. & J. Presl | | |
| 84 [136]. Vitaceae Juss. | **Vitaceae**<br>**Leeaceae** | as IFV<br>as IFV |
| **Zygophyllales** Link | | |
| 85 [138]. **Zygophyllaceae** R. Br. | as APG IV | as APG IV |
| **Fabales** Bromhead | | |
| 86 [140]. Fabaceae Lindl. | **Ceasalpiniaceae**<br>**Mimosaceae** | as APG IV |
| 87 [141]. Surianaceae Arn. | not recognized | Simaroubaceae: *Suriana* |
| 88 [142]. **Polygalaceae** Hoffmanns. & Link | as APG IV | as APG IV |
| **Rosales** Bercht. & J. Presl | | |
| 89 [143]. Rosaceae Juss. | Rosaceae: excl. *Chryso-balanus, Parinari* | as APG IV |
| 90 [146]. **Elaeagnaceae** Juss. | as APG IV | as APG IV |
| 91 [147]. **Rhamnaceae** Juss. | as APG IV | as APG IV |
| 92 [148]. Ulmaceae Mirb. | **Ulmaceae**: *Ulmus* | as APG IV |
| 93 [149]. **Cannabaceae** Martinov. | as APG IV | as APG IV |
| 94 [150]. **Moraceae** Gaudich. | as APG IV | as APG IV |
| 95 [151]. **Urticaceae** Juss. | as APG IV | as APG IV |
| **Fagales** Engl. | | |
| 96 [153]. **Fagaceae** Dumort. | as APG IV | as APG IV |
| 97 [154]. **Myricaceae** Rich. ex Kunth | as APG IV | as APG IV |
| 98 [155]. Juglandaceae DC. Ex Perleb | **Juglandaceae**<br>**Rhoipteleaceae** | as IFV<br>as IFV |
| 99 [156]. **Casuarinaceae** R. Br. | as APG IV | as APG IV |
| 100 [158]. **Betulaceae** Gray | as APG IV | as APG IV |
| **Cucurbitales** Juss. ex Bercht. & J. Presl | | |
| 101 [160]. **Anisophylleaceae** Ridl. | as APG IV | as APG IV |
| 102 [163]. **Cucurbitaceae** Juss. | as APG IV | as APG IV |
| 103 [165]. **Datiscaceae** Dumort. | as APG IV | as APG IV |
| 104 [166]. **Begoniaceae** C. Agardh | as APG IV | as APG IV |
| **[COM-clade; placement uncertain]** | | |
| **Celastrales** Link | | |
| 105 [168]. Celastraceae R. Br. | **Celastraceae**<br>not recognized | as IFV<br>Siphonodontataceae |
| **Oxalidales** Bercht. & J. Presl | | |
| 106 [170]. **Connaraceae** R. Br. | as APG IV | as APG IV |
| 107 [171]. **Oxalidaceae** R. Br. | as APG IV | as APG IV |
| 108 [173]. **Elaeocarpaceae** Juss. | as APG IV | as APG IV |
| **Malpighiales** Juss. ex Bercht. & J. Presl | | |
| 109 [179]. **Rhizophoraceae** Pers. | as APG IV | as APG IV |
| 110 [180]. **Erythroxylaceae** Kunth | as APG IV | as APG IV |
| 111 [181]. **Ochnaceae** DC. | as APG IV | as APG IV |
| 112 [182]. Bonnetiaceae L. Beauvis. ex Nakai | not recognized | as APG IV |
| 113 [183]. Clusiaceae Lindl. | **Clusiaceae**: *Garcinia* | as APG IV |
| 114 [184]. Calophyllaceae J. Agardh | Clusiaceae: *Calophyllym, Mesua* | Clusiaceae: *Calophyllym, Mesua, Kayea* |

| | | |
|---|---|---|
| 115 [185]. **Podostemaceae** Rich. ex Kunth | as APG IV | as APG IV |
| 116 [186]. Hypericaceae Juss. | Clusiaceae: *Cratoxylum, Hypericum* | as APG IV |
| 117 [189]. Putranjivaceae Meisn. | **Euphorbiaceae**: *Drypetes* | as APG IV |
| 118 [191]. **Elatinaceae** Dumort. | as APG IV | as APG IV |
| 119 [192]. **Malpighiaceae** Juss. | as APG IV | as APG IV |
| 120 [195]. **Dichapetalaceae** Baill. | as APG IV | as APG IV |
| 121 [197]. Chrysobalanaceae R. Br. | Rosaceae: *Chrysobalanus, Parinari* | as APG IV |
| 122 [199]. Achariaceae Harms | Flacourtiaceae: *Hydnocarpus* | Flacourtiaceae: *Gynocardia, Hydnocarpus* |
| 123 [200]. **Violaceae** Batsch | as APG IV | as APG IV |
| 124 [202]. Passifloraceae Juss. ex Roussel | **Passifloraceae**<br>**Turneraceae** | as IFV<br>as IFV |
| 125 [204]. Salicaceae Mirb. | Flacourtiaceae: excl. *Hydnocarpus*<br>**Salicaceae** | Flacourtiaceae: excl. *Gynocardia, Hydnocarpus*<br>as IFV |
| 126 [206]. Rafflesiaceae Dumort. | **Rafflesiaceae**: *Sapria* | as APG IV |
| 127 [207]. Euphorbiaceae Juss. | Euphorbiaceae: *Acalypha, Alchornea, Aleurites, Baliospermum, Blachia, Chaetocarpus, Cladogynos, Claoxylon, Cleidiocarpon, Cleidion, Cnesmone, Codiaeum, Croton, Dalechampia, Duetzianthus, Dimorphocalyx, Endospermum, Epiperinus, Erismanthus, Euphorbia, Excoecaria, Hevea, Homonoia, Hura, Jatropha, Lasiococca, Macaranga, Mallotus, Manihot, Melanolepis, Ostodes, Pachystylidium, Pedilanthus, Ricinus, Strophioblachia, Sumbaviopsis, Suregada, Trevia, Trigonostemon, Vernicia.* | Euphorbiaceae: *Acalypha, Alchornea, Aleurites, Baliospermum, Blachia, Chaetocarpus, Cladogynos, Claoxylon, Cleidiocarpon, Cleidion, Cnesmone, Codiaeum, Croton, Dalechampia, Duetzianthus, Dimorphocalyx, Endospermum, Epiperinus, Erismanthus, Euphorbia, Excoecaria, Hevea, Homonoia, Hura, Jatropha, Koilodepas, Lasiococca, Macaranga, Mallotus, Manihot, Melanolepis, Mercurialis, Ostodes, Pachystylidium, Pedilanthus, Ricinus, Strophioblachia, Sumbaviopsis, Suregada, Trevia, Trigonostemon, Vernicia.* |
| 128 [208]. Linaceae DC. ex Perleb. | **Linaceae**<br>Sabiaceae: *Hugonia, Indoroucheria* | as IFV<br>Hugoniaceae |
| 129 [209]. **Ixonanthaceae** Planch. ex Miq. | as APG IV | as APG IV |
| 130 [211]. Phyllanthaceae Martinov | **Euphorbiaceae**: *Aporosa, Actephila, Antidesma, Aporosa, Baccaurea, Bischofia, Breynia, Bridelia, Cleistanthus, Flueggea, Glochidion,* | as IFV |

| | | |
|---|---|---|
| | *Hymenocardia, Leptopus, Margaritaria, Phyllanthus, Sauropus* | |
| **Geraniales** Juss. ex Bercht. & J. Presl | | |
| 131 [212]. **Geraniaceae** Juss. | as APG IV | as APG IV |
| **Myrtales** Juss. ex Bercht. & J. Presl | | |
| 132 [214]. **Combretaceae** R. Br. | as APG IV | as APG IV |
| 133 [215]. Lythraceae J. St.-Hil. | **Lythraceae**<br>**Punicaceae**<br>**Sonneratiaceae**<br>**Trapaceae** | as IFV<br>as IFV<br>as IFV<br>as IFV |
| 134 [218]. **Myrtaceae** Juss. | as APG IV | as APG IV |
| 135 [219]. **Melastomataceae** Juss. | as APG IV | as APG IV |
| 136 [220]. **Crypteroniaceae** A. DC. | as APG IV | as APG IV |
| **Crossosomatales** Takht. ex Reveal | | |
| 137 [226]. Staphyleaceae Martinov. | **Staphyleaceae**: excl. *Tapiscia* | as IFV |
| 138 [228]. **Stachyuraceae** J. Agardh | as APG IV | as APG IV |
| **Huerteales** Doweld | | |
| 139 [233]. Tapisciaceae Takht.<br>140 [234]. Dipentodontaceae Merr. | **Staphyleaceae**: *Tapiscia*<br>not recognized | as IFV<br>Dipentodontaceae |
| **Sapindales** Juss. ex Bercht. & J. Presl | | |
| 141 [238]. Burseraceae Kunth | Burseraceae | Annacardiaceae: *Bursera, Canarium,* |
| 142 [239]. Anacardiaceae R. Br. | Anacardiaceae | *Dacryodes, Garuga, Protium* |
| 143 [240]. Sapindaceae Juss. | **Aceraceae**<br>**Hippocastanaceae**<br>**Sapindaceae** | as IFV<br>as IFV<br>as IFV |
| 144 [241]. Rutaceae Juss. | **Rutaceae**<br>**Simaroubaceae**: *Harrisonia* | as IFV<br>as IFV |
| 145 [242]. Simaroubaceae DC. | Simaroubaceae: excl. *Irvingia* | Simaroubaceae: excl. *Suriana* |
| 146 [243]. **Meliaceae** Juss. | as APG IV | as APG IV |
| **Malvales** Juss. ex Bercht. & J. Presl | | |
| 147 [247]. Malvaceae Juss. | **Bombacaceae**<br>**Malvaceae**<br>**Sterculiaceae**<br>**Tiliaceae** | as APG IV<br>as APG IV<br>as APG IV<br>as APG IV |
| 148 [249]. **Thymelaeaceae** Juss. | as APG IV | as APG IV |
| 149 [250]. Bixaceae Kunth | **Bixaceae**<br>**Cochlospermaceae** | as APG IV<br>as APG IV |
| 150 [253]. **Dipterocarpaceae** Blume | as APG IV | as APG IV |
| **Brassicales** Bromhead | | |
| 151 [254]. Akaniaceae Stapf. | **Bretschneideraceae** | as IFV |
| 152 [255]. **Tropaeolaceae** Juss. ex DC. | as APG IV | as APG IV |
| 153 [256]. **Moringaceae** Martinov | as APG IV | as APG IV |
| 154 [257]. **Caricaceae** Dumort. | as APG IV | as APG IV |
| 155 [262]. **Salvadoraceae** Lindl | as APG IV | as APG IV |
| 156 [267]. Resedaceae Martinov | Capparaceae: *Neothorelia, Stixis, Tirania* | Capparaceae: *Stixis, Tirania* |

| | | |
|---|---|---|
| 157 [268]. Capparaceae Juss. | **Capparaceae**: excl. *Cleome* | as IFV |
| 158 [269]. Cleomaceae Bercht. & J. Presl | **Capparaceae**: *Cleome* | as IFV |
| 159 [270]. **Brassicaceae** Burnett | as APG IV | as APG IV |
| **SUPERASTERIDS** | | |
| **Santalales** R. Br. ex Bercht. & J. Presl | | |
| 160 [273]. 'Olacaceae' R. Br. | **Olacaceae**<br>not recognized | as IFV<br>Erythropalaceae |
| 161 [274]. **Opiliaceae** Veleton | as APG IV | as APG IV |
| 162 [275]. **Balanophoraceae** Rich. | as APG IV | as APG IV |
| 163 [276]. 'Santalaceae' R. Br. | **Santalaceae**<br>Loranthaceae: *Ginalloa, Korthalsella, Viscum* | as IFV<br>Viscaceae |
| 164 [278]. Schoepfiaceae Blume | Olacaceae: *Schoepfia* | as APG IV |
| 165 [279]. Loranthaceae Juss. | **Loranthaceae** | as IFV<br>Saxifragaceae: *Pilostigma* |
| **Caryophyllales** Juss. ex Bercht. & J. Presl | | |
| 166 [281]. **Tamaricaceae** Link | as APG IV | as APG IV |
| 167 [282]. **Plumbaginaceae** Juss. | as APG IV | as APG IV |
| 168 [283]. **Polygonaceae** Juss. | as APG IV | as APG IV |
| 169 [284]. **Droseraceae** Salisb. | as APG IV | as APG IV |
| 170 [285]. **Nepenthaceae** Dumort. | as APG IV | as APG IV |
| 171 [288]. **Ancistrocladaceae** Planch. ex Walp. | as APG IV | as APG IV |
| 172 [295]. **Caryophyllaceae** Juss. | as APG IV | as APG IV |
| 173 [297]. Amaranthaceae Juss. | **Amaranthaceae**<br>**Chenopodiaceae** | as IFV<br>as IFV |
| 174 [303]. Gisekiaceae Nakai | Aizoaceae: *Gisekia* | Molluginaceae: *Gisekia* |
| 175 [304]. Aizoaceae Martinov | **Aizoaceae**<br>not recognized | as IFV<br>Tetragoniaceae |
| 176 [305]. **Phytolaccaceae** R. Br. | as APG IV | as APG IV |
| 177 [308]. **Nyctaginaceae** Juss. | as APG IV | as APG IV |
| 178 [309]. Molluginaceae Bartl. | **Molluginaceae**: *Glinus, Mollugo* | as IFV |
| 179 [312]. **Basellaceae** Raf. | as APG IV | as APG IV |
| 180 [314]. Talinaceae Doweld | **Portulacaceae**: *Talinum* | as IFV |
| 181 [315]. **Portulacaceae** Juss. | as APG IV | as APG IV |
| 182 [317]. **Cactaceae** Juss. | as APG IV | as APG IV |
| **ASTERIDS** | | |
| **Cornales** Link | | |
| 183 [318]. Nyssaceae Juss. ex Dumort. | **Nyssaceae**<br>Cornaceae: *Mastixia*<br>not recognized | as IFV<br>Mastixiaceae<br>Davidiaceae |
| 184 [320]. **Hydrangeaceae** Dumort. | Hydrangeaceae: *Dichroa, Hydrangea , Pliostegia, Schizophragma* | Saxifragaceae: *Dichroa, Hydrangea* |
| 185 [324]. Cornaceae Bercht. & J. Presl | **Alangiaceae**<br>Cornaceae: *Cornus* | as IFV<br>Cornaceae |
| **Ericales** Bercht. & J. Presl | | |
| 186 [325]. **Balsaminaceae** A. Rich. | as APG IV | as APG IV |
| 187 [329]. **Polemoniaceae** Juss. | as APG IV | as APG IV |

| | | |
|---|---|---|
| 188 [330]. **Lecythidaceae** A. Rich. | as APG IV | as APG IV |
| 189 [332]. Pentaphylacaceae Engl. | **Pentaphylacaceae**<br>Theaceae: *Adinandra, Anneslea, Eurya, Ternstroemia* | as IFV<br>Theaceae: *Adinandra, Anneslea, Eurya* |
| 190 [333]. **Sapotaceae** Juss. | as APG IV | as APG IV |
| 191 [334]. **Ebenaceae** Gurke | as APG IV | as APG IV |
| 192 [335]. Primulaceae Batsch ex Borkh. | **Myrsinaceae**<br>**Primulaceae** | as IFV<br>as IFV |
| 193 [336]. Theaceae Mirb. | Theaceae: excl. *Adinandra, Anneslea, Eurya, Temstroemia* | Theaceae: excl. *Adinandra, Anneslea, Eurya* |
| 194 [337]. **Symplocaceae** Desf. | as APG IV | as APG IV |
| 195 [338]. Diapensiaceae Lindl. | not recognized | as APG IV |
| 196 [339]. **Styracaceae** DC. & Spreng. | as APG IV | as APG IV |
| 197 [342]. **Actinidiaceae** Gilg & Werderm. | as APG IV | as APG IV |
| 198 [343]. **Clethraceae** Klotzsch | as APG IV | as APG IV |
| 199 [345]. Ericaceae Juss. | **Ericaceae**<br>**Epacridaceae**<br>not recognized | as IFV<br>as IFV<br>Pyrolaceae |
| 200 [346]. Mitrastemonaceae Makino [placement in order unclear] | **Rafflesiaceae**: *Mitrastemon* | as IFV |
| # **Icacinales** Tiegh. | | |
| 201 [348]. Icacinaceae Miers | **Icacinaceae**: excl. *Gomphandra, Gonocaryum* | as IFV |
| **Garryales** Mart. | | |
| 202 [350]. **Eucommiaceae** Engl. | as APG IV | as APG IV |
| 203 [351]. Garryaceae Lindl. | Cornaceae: *Aucuba* | Aucubaceae |
| **Gentianales** Juss. ex Bercht. & J. Presl | | |
| 204 [352]. Rubiaceae Juss. | **Rubiaceae**: excl. *Carlemannia* | as IFV |
| 205 [353]. Gentianaceae Juss. | **Gentianaceae**<br>**Potaliaceae** | as IFV<br>as IFV |
| 206 [354]. Loganiaceae R. Br. ex Mart. | **Loganiaceae**: *Mitrasacme, Mitreola, Strychnos* | as IFV |
| 207 [356]. Apocynaceae Juss. | **Apocynaceae**<br>**Asclepiadaceae** | as IFV<br>as IFV |
| **Boraginales** Juss. ex Bercht. & J. Presl | | |
| 208 [357]. **Boraginaceae** Juss. | as APG IV | as APG IV |
| **Solanales** Juss. ex Bercht. & J. Presl | | |
| 209 [359]. Convolvulaceae Juss. | **Convolvulaceae**<br>not recognized | as IFV<br>Cuscutaceae |
| 210 [360]. **Solanaceae** Juss. | as APG IV | as APG IV |
| 211 [362]. **Sphenocleaceae** T. Baskerv. | as APG IV | as APG IV |
| 212 [363]. **Hydroleaceae** R. Br. | as APG IV | as APG IV |
| **Lamiales** Romhead | | |
| 213 [365]. Carlemanniaceae Airy Shaw | **Rubiaceae**: *Carlemannia*<br>**Caprifoliaceae**: *Silvianthus* | as IFV<br>as IFV |

| | | |
|---|---|---|
| 214 [366]. **Oleaceae** Hoffmanns. & Link | as APG IV | as APG IV |
| 215 [369]. **Gesneriaceae** Rich. & Juss. | as APG IV | as APG IV |
| 216 [370]. Plantaginaceae Juss. | **Plantaginaceae** | as IFV |
| | Scrophulariaceae: *Angelonia* | Scrophulariaceae: *Adenosma, Angelonia, Antirrhinum, Bacopa, Digitalis, Limnophila, Russelia, Scoparia, Stemodia, Veronica* |
| | **Callitrichiaceae** | as IFV |
| 217 [371]. Scrophulariaceae Juss. | **Buddlejaceae** | as IFV |
| | **Myoporaceae** | as IFV |
| | **Scrophulariaceae**: *Scrophularia* | as IFV |
| 218 [373]. Linderniaceae Borsch et al. | **Scrophulariaceae**: *Legazpia, Linderna, Picria, Pierranthus, Torenia* | as IFV |
| 219 [375]. **Martyniaceae** Horan. | as APG IV | as APG IV |
| 220 [376]. **Pedaliaceae** R. Br. | as APG IV | as APG IV |
| 221 [377]. Acanthaceae Juss. | **Acanthaceae** | as IFV |
| | **Verbenaceae**: *Avincennia* | as IFV |
| 222 [378]. **Bignoniaceae** Juss. | as APG IV | as APG IV |
| 223 [379]. **Lentibulariaceae** Rich. | as APG IV | as APG IV |
| 224 [382]. Verbenaceae J. St. Hil. | **Verbenaceae**: *Duranta, Lantana, Phyla, Stachtarpheta, Verbena* | as IFV |
| 225 [383]. Lamiaceae Martinov | **Lamiaceae** | as IFV |
| | **Verbenaceae**: *Callicarpa, Caryopteris, Clerodendron, Congea, Garrettia, Gmelina, Hymenopyramis, Karomia, Prema, Schnabelia, Sphenodesme, Tectona, Teijsmanniodendron, Tsoongia, Vitex* | as IFV |
| 226 [384]. Mazaceae Reveal | **Scrophulariaceae**: *Mazus* | as IFV |
| 227 [385]. Phrymaceae Schauer | **Scrophulariaceae**: *Mimulus, Mazus, Microcarpaea* | as IFV |
| 228 [386]. Paulowniaceae Nakai | **Scrophulariaceae**: *Paulownia* | as IFV |
| 229 [387]. Orobanchaceae Vent. | **Orobanchaceae** | as IFV |
| | **Scrophulariaceae**: *Alectra, Brandisia, Centranthera, Lindendergia, Pedicularis, Rehmannia, Sopubia, Wightia* | as IFV |
| **Aquifoliales** Senft | | |

| | | |
|---|---|---|
| 230 [388]. Stemonuraceae Karehed | **Icacinaceae**: *Gomphandra* | as IFV |
| 231 [389]. Cardiopteridaceae Blume | **Cardiopteridaceae**<br>**Icacinaceae**: *Gonocaryum* | as IFV<br>as IFV |
| 232 [391]. Helwingiaceae Decne. | Cornaceae: *Helwingia* | as APG IV |
| 233 [392]. **Aquifoliaceae** Bercht. & J. Presl | as APG IV | as APG IV |
| **Asterales** Link | | |
| 234 [394]. Campanulaceae Juss. | **Campanulaceae**: excl. *Pentaphragma*<br>**Lobeliaceae** | as IFV<br>as IFV |
| 235 [395]. Pentaphragmataceae J. Agardh | **Campanulaceae**: *Pentaphragma* | as IFV |
| 236 [396]. **Stylidiaceae** R. Br. | as APG IV | as APG IV |
| 237 [400]. **Menyanthaceae** Dumort. | as APG IV | as APG IV |
| 238 [401]. **Goodeniaceae** R. Br. ex Rich. | as APG IV | as APG IV |
| 239 [403]. **Asteraceae** Bercht. & J. Presl | as APG IV | as APG IV |
| **Escalloniales** Link | | |
| 240 [404]. Escallonoiaceae R. Br. ex Dumort. | Saxifragaceae: *Polyosma* | Escallonoiaceae: *Polyosma* |
| **Dipsacales** Juss. ex Bercht. & J. Presl | | |
| 241 [408]. Adoxaceae E. Mey. | **Caprifoliaceae**: *Sambucus, Viburnum* | as IFV |
| 242 [409]. Caprifoliaceae Juss. | Caprifoliaceae: *Lonicera*<br>**Dipsacaceae**<br>**Valerianaceae** | Caprifoliaceae: *Abelia, Lonicera*<br>as IFV<br>as IFV |
| **Apiales Nakai** | | |
| 243 [411]. Torricelliaceae Hu | Cornaceae: *Torricellia* | as APG IV |
| 244 [413]. Pittosporaceae R. Br. | Pittosporaceae: excl. *Penthorum* | as APG IV |
| 245 [414]. Araliaceae Juss. | **Araliaceae**<br>**Apiaceae**: *Hydrocotyle* | as IFV<br>as IFV |
| 246 [416]. Apiaceae Lindl. | **Apiaceae**: excl. *Hydrocotyle* | as IFV |

***Note:*** Numbers in square brackets are those of the linear Angiosperm Phylogeny Group of APG IV (2016).
